# Multi-omics landscape of childhood simple obesity: novel insights into pathogenesis and biomarkers discovery

**DOI:** 10.1186/s13578-024-01322-5

**Published:** 2024-11-28

**Authors:** Yi Ren, Peng Huang, Lu Zhang, Yufen Tang, Siyi He, HaiDan Li, XiaoYan Huang, Yan Ding, Lingjuan Liu, Liqun Liu, Xiaojie He

**Affiliations:** 1 Department of Pediatrics, The Second Xiangya Hospital of Central South University, Changsha, Hunan 410011 China; 2 Children’s Brain Development and Brain Injury Research Office, The Second Xiangya Hospital of Central South University, Changsha, 410011 China; 3 Department of Pediatrics, Haikou Hospital of the Maternal and Child Health, Haikou, 570100 China; 4 Department of Pediatrics, Hainan Modern Women and Children’s. Medical, Haikou, 570100 China; 5 Department of Anesthesiology, Hainan General Hospital, Haikou, Hainan, 570311 China; 6 Hainan Affiliated Hospital of Hainan Medical University, Haikou, Hainan, 570311 China; 7 Department of Pediatrics, Hainan Women and Children’s Medical Center, Hainan, 570100 China; 8 Department of Dermatology, Hospital of Hainan Medical University, Haikou, Hainan, 570311 China; 9 Laboratory of Pediatric Nephrology, Department of Pediatrics, The Second Xiangya Hospital of Central South University, Changsha, 410011 China

**Keywords:** Obesity, Childhood, Transcriptomics, Metabolomics, 16S rDNA, And multi-omics

## Abstract

**Background:**

The increasing incidence of childhood obesity annually has led to a surge in physical and mental health risks, making it a significant global public health concern. This study aimed to discover novel biomarkers of childhood simple obesity through integrative multi-omics analysis, uncovering their potential connections and providing fresh research directions for the complex pathogenesis and treatment strategies.

**Methods:**

Transcriptome, untargeted metabolome, and 16 S rDNA sequencing were conducted on subjects to examine transcripts, metabolites in blood, and gut microflora in stool.

**Results:**

Transcriptomic analysis identified 599 differentially expressed genes (DEGs), of which 25 were immune-related genes, and participated in immune pathways such as antimicrobial peptides, neutrophil degranulation, and interferons. The optimal random forest model based on these genes exhibited an AUC of 0.844. The metabolomic analysis examined 71 differentially expressed metabolites (DEMs), including 12 immune-related metabolites. Notably, lauric acid showed an extremely strong positive correlation with BMI and showed a good discriminative power for obesity (AUC = 0.82). DEMs were found to be significantly enriched in four metabolic pathways, namely “Aminoacyl-tRNA biosynthesis”, “Valine leucine and isoleucine biosynthesis, and Glycine”, “Serine and threonine metabolism”, and “Biosynthesis of unsaturated fatty acids”. Microbiome analysis revealed 12 differential gut microbiotas (DGMs) at the phylum and genus levels, with *p_Firmicutes* dominating in the obese group and *g_Escherichia-Shigella* in the normal group. Subsequently, a Random Forest model was developed based on the DEMs, immune-related DEGs, and metabolites with an AUC value of 0.912. The 14 indicators identified by this model could potentially serve as a set of biomarkers for obesity. The analysis of the inter-omics correlation network found 233 pairs of significant correlations. DEGs BPIFA1, BPI, and SAA1, DEMs Dimethy(tetradecyl)amine, Deoxycholic acid, Pathalic anhydride, and DL-Alanine, and DGMs *g_Intestinimonas* and *g_Turicibacter* showed strong connectivity within the network, constituting a large proportion of interactions.

**Conclusion:**

This study presents the first comprehensive description of the multi-omics characteristics of childhood simple obesity, recognizing promising biomarkers. Immune-related markers offer a new perspective for researching the immunological mechanisms underlying obesity and its associated complications. The revealed interactions among these biomarkers contribute to a deeper understanding the intricate biological regulatory networks associated with obesity.

**Supplementary Information:**

The online version contains supplementary material available at 10.1186/s13578-024-01322-5.

## Introduction

Obesity, characterized by abnormal or excessive fat accumulation, has emerged as a global epidemic. Of particular concern is the escalating prevalence among children, with over 379 million children and adolescents affected by overweight or obesity [1], which constitutes an enormous public health crisis. Childhood obesity not only threatens the physical health of children, leading to conditions such as impaired left ventricular diastolic function, early puberty, type 2 diabetes mellitus (T2DM), and asthma, but also exerts a lasting influence on cognitive abilities, mental health, and social adaptation, thereby significantly diminishing their overall quality of life [2–4]. Moreover, childhood obesity is recognized as an independent risk factor for adult obesity, increasing the likelihood of developing metabolic syndrome, cancer, autoimmune diseases, and other conditions in adulthood in the absence of timely intervention [5, 6]. Nevertheless, the precise molecular mechanisms underlying the occurrence and development of childhood simple obesity have yet to be fully elucidated.

High-throughput transcriptome sequencing could identify the differentially expressed genes (DEGs) by providing a wealth of information on gene expression in disease states, contributing to finding novel biomarkers associated with disease. Furthermore, gene function annotation and pathway analysis could enhance our understanding of the major pathways implicated in disease onset and progression. Metabolomics facilitates the detection of small molecule metabolites in organisms, providing insights into how genetics, environmental factors, or gut microbiota affect host biochemical processes [7]. Through untargeted metabolomics, we could gain a comprehensive understanding of the composition and alterations of plasma metabolites among children with simple obesity and reveal changed biochemical pathways during the obesity state.

Genetic factors play a critical role in the pathogenesis of obesity, and the environmental factors, lifestyle, and gut microbiota also contribute significantly. As an essential ecosystem within the human body, the intestinal microbiome could complement the functions of the human genome through its diverse metabolic properties. The gut microbiota serves as a bridge connecting host genetics, metabolism, immune responses, and environmental influences, and dysregulation of the gut microbiota has been closely related to the initiation and progression of multiple diseases [8]. However, the precise characteristics of gut microbiota in children with simple obesity remain controversial. This study employs 16 S rDNA sequencing to explore the core microbiota and their functional features in childhood simple obesity at our medical center.

Additionally, obesity is considered a condition characterized by persistent low-grade inflammation, the local site of inflammation of the adipose tissue and its induced systemic inflammation is a major driver of obesity [9, 10]. Importantly, complex interactions between the host immune response and metabolites contribute to the exacerbation of this process. Lipid metabolites, vitamins, and amino acids, for instance, have the capacity to directly regulate the immune cell reactivity, thereby promoting inflammation responses [10–12]. Inflammatory states and inflammation reactions may alter the levels of metabolites linked to biological processes that promote obesity, such as glucose metabolism and fatty acid synthesis [13]. Therefore, it is necessary to understand this bidirectional regulation mechanism involved in obesity.

In this study, we were the first to integrate transcriptomics, untargeted metabolomics, and gut microbiome aiming to uncover potential biomarkers and molecular characteristics relevant to the prevention and treatment of childhood simple obesity. Meanwhile, we first use “immunity” as a connecting element to explore the interactions among host genes, metabolites, and gut microbiota, providing new perspectives and theoretical foundations for comprehending the immune regulatory network involved in the pathogenesis of obesity.

## Materials and methods

### Study population

This cross-sectional study enrolled a total of 73 participants, consisting of 40 obese children and 33 healthy controls, with ages ranging 6–14 years, from the Department of Pediatrics at the Maternal and Child Health Hospital of Haikou, Hainan Province, China. General and clinical data, including the levels of vitamin D (Vit D), triglycerides (TG), and uric acid (UA) in the blood, were collected from all subjects. And body mass index (BMI) was calculated using the formula weight (kg)/ (height[m]^2^). Given variations in ethnicity, age, and gender, this study employed specific BMI thresholds for Chinese children. Children falling within a BMI ≥ 97th percentile for their age and gender were classified as obese, while those within a BMI between the 15th and 85th percentiles were classified as the normal group. Individuals with other diseases such as metabolic disorders, endocrine disorders, or secondary obesity due to other diseases or glucocorticoid therapy were excluded. Additionally, individuals who had undergone gastrointestinal surgery or had taken antibiotics, hormones, or probiotics within the previous three months were also excluded from the study. This research project has been approved by the Medical Ethics Committee of Haikou Hospital of the Maternal and Child Health (Approval number: [2019]01001). Furthermore, written informed consent was provided by the parents or legal guardians of all participants.

### Transcriptome sequencing and analysis

#### Whole transcriptome sequencing and data processing

Totaling 2–3 ml of peripheral blood samples were collected in EDTA tubes in the fasting state of individuals. Plasma was obtained by centrifugation of the EDTA blood and stored at -80°C. Total RNA was extracted from the peripheral blood samples using the PX Blood RNA Kit (200) (Cat#R1057-02, Omega) following the manufacturer’s instructions. After checking quality using Agilent Bioanalyzer 2100 (Agilent Technologies, Santa Clara, CA, USA), the extracted RNA samples were further purified using the RNAClean XP Kit (Cat#A63987, USA) and RNase-Free DNase Set (Cat#79254, QIAGEN, Germany). Subsequently, the constructed cDNA libraries were sequenced bidirectionally using the Illumina Hiseq 2500 platform. Only sequences with at least 85% of the bases with a quality score above 20 were used for sequencing library construction. Raw reads were filtered through seqtk (https://github.com/lh3/seqtk) to remove the adaptor sequences, reads bases with lower quality than 20 at the 3’ end, reads shorter than 25 bp, and ribosomal RNA reads. Then, clean reads were preserved for subsequent assays and aligned to the GRCh38 human genome using HISAT2. Gene expression values were standardized to compare gene expression levels across different genes and samples. Specifically, the Fragments were quantified for each mapped gene by Stringtie software v1.3.0 and the data were normalized using the Trimmed Mean of M (TMM) values method to calculate the Fragments Per Kilobase of exon model per Million mapped reads (FPKM) for each gene. Pearson correlation analysis was performed based on the FPKM quantification results, and principal coordinate analysis (PCoA) was utilized based on the Bray-Curtis dissimilarity, to assess the experiment’s reproducibility.

#### Differentially expressed gene analysis

The raw gene expression data was initially analyzed using edgeR software package to obtain *P*-values. Subsequently, according to the FPKM values, the fold-change (FC) in expression for each gene between the two groups was calculated to compute the Log_2_FC. Genes with |log_2_FC| >1 and a *P*-value < 0.05 were classified as DEGs.

#### Functional analysis of DEGs

Function and pathway enrichment of DEGs were analyzed by Gene Ontology (GO) and Kyoto Encyclopedia of Genes and Genomes (KEGG) pathway databases.

#### Identification and functional analysis of immune-related DEGs

The InnateDB database (https://www.innatedb.com), containing over 18,000 molecular interactions related to innate immunity, provides a comprehensive resource for innate immunity component molecules, their biological pathways, and networks. In this study, we downloaded the human innate immune response genes from InnateDB to identify immune-related DEGs associated with obesity. Leveraging these immune-related DEGs, a random forest model was constructed to recognize the immune-related DEGs contributing to the development of obesity. The biological functional analysis of these genes was performed through the Metascape online platform (https://metascape.org), with specific parameters set as *P*-value less than 0.01, minimum count of 3, and enrichment factor exceeding 1.5.

#### Construction of protein-protein interaction (PPI) network associated with immune-related DEGs

To explore the inner connections among immune-related DEGs, we utilized the String database (https://cn.string-db.org) to construct a PPI network associated with immune-related DEGs and visualized the results via Cytoscape software.

### Untargeted metabolomic sequencing and analysis

#### Blood metabolite extraction

Peripheral venous blood samples (2–3 ml) from subjects were collected in EDTA tubes and centrifuged at 3000 rpm and 4 °C for 10 min. Then the plasma obtained was immediately stored at -80 °C. Afterward, 100ul of plasma was put in an EP tube, followed by the addition of 400 µl of methanol and vortexed for 30 s, incubation at -40 °C for 1 h, vertexing for another 30 s, and further incubation at 4 °C for 0.5 h. Subsequently, samples were centrifuged at 4 °C and 12,000 rpm for 15 min. Finally, 200ul of the supernatant was mixed with 5 µl of chlorphenamine maleate (1 mg/ml) and tested on the computer. A pooled quality control (QC) sample was prepared by mixing equal volumes from each plasma sample.

#### lc-MS/MS analysis

To identify the metabolomic features of blood samples from subjects, this study employed an ultra-performance liquid chromatography system with quadrupole-time-of-flight mass spectrometry (UPLC-QTOF/MS) for untargeted metabolomic analysis. All samples were separated by the ACQUITY UPLC HSS T3 column. The chromatographic conditions for detection included an autosampler temperature of 4℃, a column oven temperature of 45℃, a flow rate of 0.3 ml/min, and a sample injection volume of 6µL. The mobile phase comprised of mobile phase A (0.05% formic acid and water) and mobile phase B (acetonitrile). The procedure is detailed in the section of Supplementary Table 1.

Mass spectrometry (MS) was operated in both positive and negative ion modes with the electrospray ionization (ESI) technique. The MS detection parameters are detailed in Supplementary Table 2. Data were acquired in full scan mode (scan range of m/z 70-1050 and resolution of 70,000) and data-dependent MS/dd-MS2 scanning (TopN = 10 and resolution of 17,500). Higher energy collision dissociation mode was employed for targeted spectra acquisition.

#### Identification, pathway enrichment analysis, and classification performance of differentially expressed metabolites (DEMs)

The raw data obtained through mass spectrometry detection were processed using Compound Discoverer 3.1 software for identification and quantification by matching with the mzCloud, ChemSpider, and MassBank databases, followed by quality control procedures. Subsequently, Metabolites were annotated using the HMDB, KEGG, and Medline databases. The differences in metabolite between the obesity group and the normal group were calculated by the multivariate statistical analysis, including orthogonal partial least squares discriminant analysis (OPLS-DA). Metabolites with VIP > 1 and *P*-value < 0.05 were recognized as significantly differentially expressed metabolites (DEMs). The KEGG database was used to find enriched metabolic signaling pathways associated with DEMs. The performance of DEMs in classification was assessed using the area under the receiver-operating characteristic (ROC) curve (AUC) analysis.

### 16S rDNA sequencing and analyzing of gut microbiome

To determine the composition, of the microbial community, we used 16 S rDNA sequencing. Over 500 mg of fresh stool samples from subjects using a sterile scoop were collected in sterile EP tubes, and then stored at -80℃ until further analysis. The microbial genomic DNA was extracted from stool samples using the fecal genomic DNA kit (CWBIO, China). DNA purity and concentration were assessed by 1% agarose gel electrophoresis following the manufacturer’s instructions. Using diluted genomic DNA as a template, V4 hypervariable region of 16 S rDNA gene was amplified using barcode-specific primers (338 F, 5′-ACTCCTACGGGAGGCAGCAG-3′ and 806R, 5′-GGACTACHVGGGTWTCTAAT-3′) under the conditions of GeneAmp 9700 systems thermocycler and TransStart Fastpfu DNA polymerase. The PCR amplification reactions for the 16 S rDNA gene were as follows: (a) denaturation at 95 °C for 3 min; (b) 29 cycles of denaturation at 95 °C for 30 s, annealing at 54 °C for 30 s and extension at 72 °C for 45 s; (c) final extension of 10 min at 72 °C. The PCR reaction system had a final volume of 20 µl. All samples were performed in triplicate. PCR products were detected on a 2% agarose gel.

Following the construction of the library, the V4 region of 16 S rDNA was sequenced on the Illumina Miseq PE300 platform (Illumina, San Diego, USA). The raw fastq 16 S rDNA sequence data was processed and analyzed using QIIME (Version 2020.2) software. For each sample, sequences with a 97% similarity threshold were clustered into operational taxonomic units (OTUs) using the USEARCH and RDP Classifier Bayes algorithm. Then OTUs representative sequences were aligned with reference sequences in the Silva database to facilitate species annotation and classification. Alpha diversity was assessed through the Chao1 index to measure microbial species richness and the Shannon index to evaluate species diversity. In parallel, Beta diversity was assessed via PCoA based on the Bray-Curtis metrics, and the differences between the two groups were determined through PERMANOVA analyses.

DGMs between the two groups at the phylum and genus levels were compared using the Wilcoxon rank-sum test (*P-* value < 0.05 was considered statistically different). Meanwhile, Linear discriminant analysis (LDA) effect size (LEfSe) was used to identify the contributions of DGMs. ROC curve analysis was conducted to assess the predictive value of DGMs. The alterations in the potential functions of the intestinal microbiota in individuals with obesity compared to those in normal individuals were performed through KEGG and COG annotations based on PICRUST2. Spearman correlation analysis was conducted to detect correlations between clinical parameters and DGMs.

### Correlation analysis among transcriptomic, metabolomic, and 16 S rDNA datasets, and construction of a multivariate linear regression model

The correlation between the expression level or abundance values of DEGs, DEMs, and DGMs was determined using Spearman correlation analysis to understand their mutual relationships. Subsequently, a multiple linear regression model was constructed using immune-related DEGs as the dependent variable, and immune-related DEMs and DGMs as independent variables.

## Results

### Transcriptome sequencing results

**Fig. 1:**
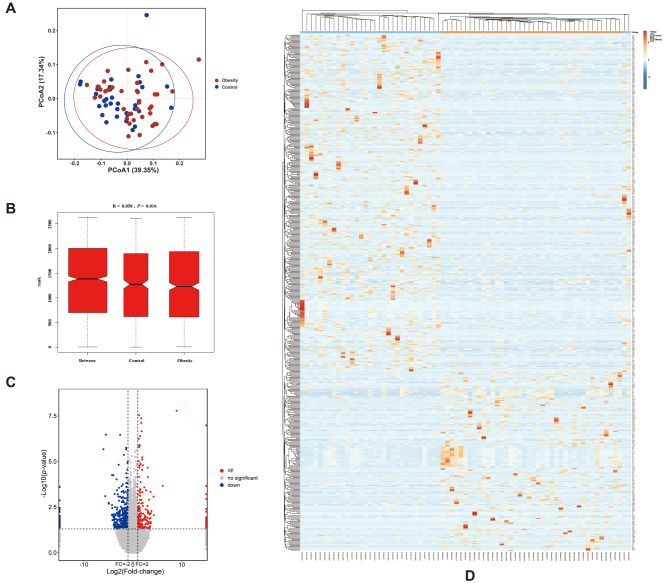
(**A**) Results of PCoA analysis based on the Bray-Curtis distance. Each point represents one sample and the distances between dots reflect differences in overall gene expression levels among samples. (**B**) Results of ANOSIM of similarities. The vertical axis of the box plot represents distance ranking, with “between” indicating between-group distances. “Control” and “Obesity” indicate their respective within-group distances. The R-value reflects the difference between groups and within groups, with a range of [-1, 1]. If *R* < 0, between-group differences are smaller than within-group differences; if *R* > 0, they are larger; if *R* = 0, they are equal. (**C**) The volcano plot shows differentially expressed genes (DEGs). The red and blue points denote significantly upregulated and downregulated DEGs, respectively. The points located on the extreme right of the y-axis solid line demonstrate that the mean expression level of genes within the normal group is zero, thereby causing the Log_2_ fold change (Log_2_FC) to approach positive infinity. Conversely, the points situated on the extreme left of the y-axis solid line indicate that the mean expression level of genes in the obese group is zero, leading to the Log_2_FC approaching negative infinity. (**D**) The heatmap of clustering of samples based on the expression levels of DEGs. A small square symbolizes a gene, with color denoting expression level. Red for high and blue for low. The top dendrogram shows sample clustering from various groups, while the left dendrogram shows the clustering analysis results of different genes from different samples

A total of 31,989 unigenes were acquired from 73 samples. Although the PCoA visualization plot shows that the normal and obese groups are not well separated (Fig. 1A), the ANOSIM similarity analysis indicates that the between-group similarity is significantly less than the within-group similarity (Fig. 1B). Even though the R value from the ANOSIM analysis is quite small (0.058), it statistically suggests that there may be a certain number of differentially expressed genes (DEGs) between the obese group and the normal control group. In fact, according to the preset screening criteria, we finally identified 599 DEGs, including 207 upregulated genes and 392 downregulated genes (Fig. 1C and Supplementary Table 3). Figure 1D presents a heatmap illustrating the expression levels of all differentially expressed genes between the two groups. The obesity group enriched a large number of downregulated DEGs compared with the control group. Subsequently, GO enrichment and KEGG pathway analyses were conducted to investigate the biological functions and pathways potentially involved by DEGs.

**Fig. 2:**
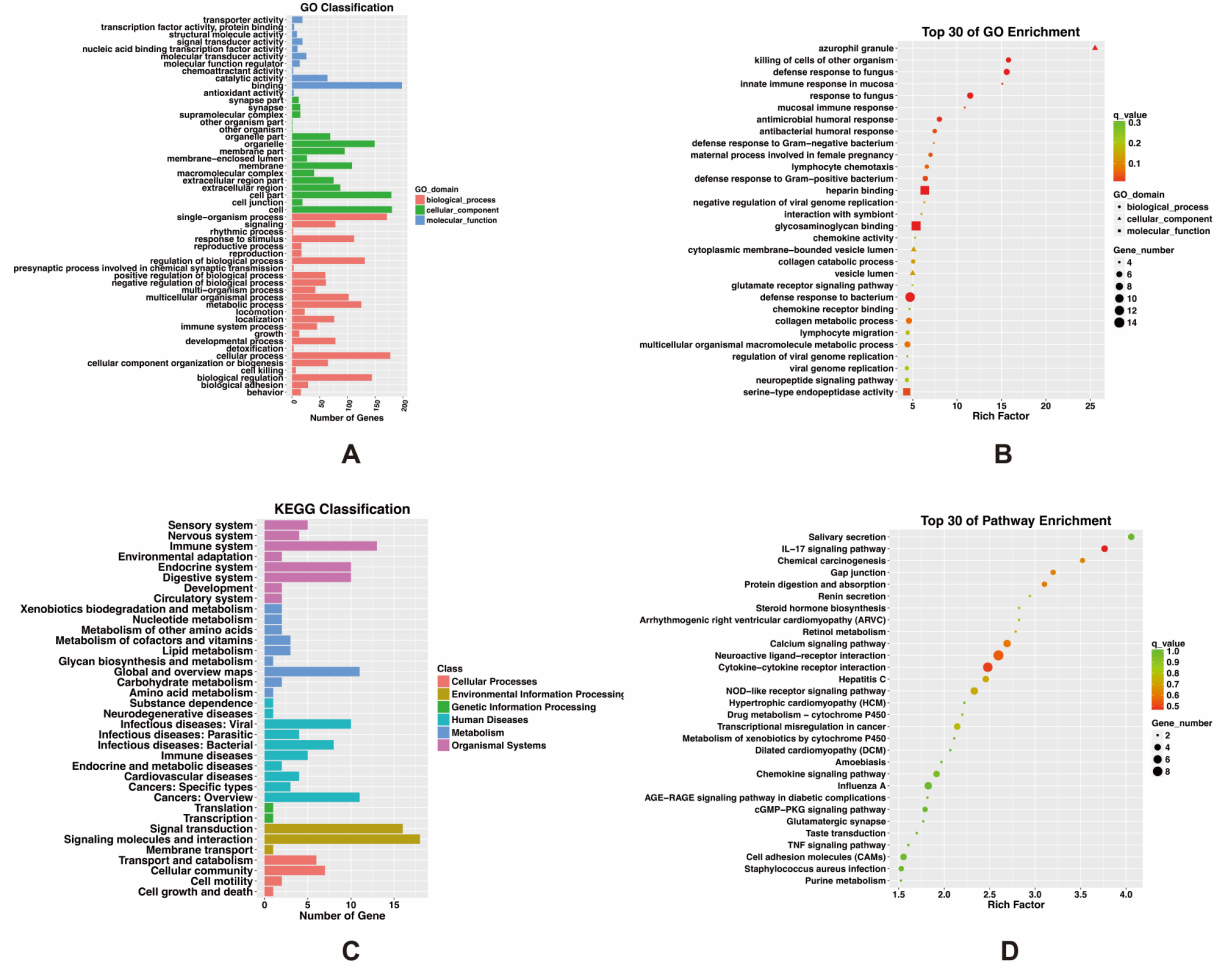
Functional annotations of differentially expressed genes. (**A**) and (**C**) show the GO and KEGG classifications of DEGs, respectively. The abscissa indicates the number of genes, while the ordinate represents the classification of GO terms and KEGG pathways, respectively. (**B**) and (**D**) show the bubble plots of GO and KEGG enrichment analyses, displaying the top 30 enriched GO terms and KEGG pathways, respectively. The larger the Rich Factor and the smaller the *q*-value, the more significant the degree of enrichment. And the size of the bubble indicates the number of enriched genes

The GO class analysis showed that the DEGs enriched in 25 biological processes, 16 cellular components, and 11 molecular functions, as detailed in Supplementary Tables 4 and Fig. 2A. Furthermore, GO enrichment analysis revealed DEGs were significantly enriched in 156 terms (*P*-value < 0.05) (Supplementary Table 4). The top 30 significantly enriched GO terms were visually depicted in Fig. 2B. Our analysis revealed that a substantial portion (over 30%) of the significantly enriched terms within the biological process classification were related to inflammatory immune responses, such as defense response to microorganisms, humoral immune response, Type I interferon signaling, and chemokine signaling. However, upregulated genes were mainly enriched in antimicrobial/antibacterial humoral response, innate immune response in the mucosa, and humoral immune response (Supplementary Fig. 1A), whereas downregulated genes were primarily enriched in terms related to chemokine and Type I interferon signaling pathway (Supplementary Fig. 1B). Previous studies have indicated a close correlation between obesity and its associated complications with the immune response, in which Type I interferons and chemokines and their receptors play crucial roles [14–18]. The findings of the GO enrichment analysis in this study further bolster this assertion, and it seems that upregulated and downregulated genes have varying impacts on the immune-related mechanisms of obesity. The significantly enriched pathway of upregulated DEGs was related to serine hydrolysis, such as serine-type endopeptidase activity, serine-type peptidase activity, and serine hydrolase activity. However, the most significantly enriched pathway for downregulated DEGs was “hormone activity,“. And downregulated DEGs were significantly enriched in pathways associated with calcium ion metabolism, such as cellular calcium ion homeostasis, calmodulin binding, calcium ion transport, etc. A prior investigation reported that serum serine levels are significantly decreased in obesity and kidney dysfunction related to obesity populations [19]. Serine has been shown to reduce serum triglycerides (TG) and cholesterol levels by suppressing the expression of genes involved in TG accumulation, thereby preventing obesity induced by high-fat diet (HFD). Therefore, in this study, the elevated expression of upregulated DEGs enriched in serine metabolism, such as ELANE, CTSG, and MMP8, could result in increased serine hydrolysis and a subsequent reduction in serine levels within the body, thereby contributing to the onset and development of obesity. On the other hand, the antagonism of the T-type calcium channel could inhibit HFD-induced weight gain, while the leakage of intracellular Ca^2+^ through the RyR2 channel could induce glucose intolerance [20, 21]. The decreased expression of downregulated DEGs enriched in Ca^2+^ metabolism, including CXCL10, CXCL13, and PDE1C, as identified in this study could play a role in obesity and its associated complications by lowering Ca^2+^ levels.

In living organisms, different genes coordinately carry out their biological functions. The identification of significantly enriched pathways of DEGs aided in the screening of the most crucial biochemical metabolism and signal transduction pathways. In this study, the KEGG classification indicated that DEGs were enriched in the immune system, endocrine system, and digestive system, and play role in the occurrence of diseases such as infectious diseases, immune diseases, and cancers (Fig. 2C). Figure 2D displayed the top 30 enriched KEGG pathways of DEGs, among which significantly enriched pathways (*P*-value < 0.05) included Salivary secretion, Neuroactive ligand-receptor interaction, IL-17 signaling pathway, Cytokine-cytokine receptor interaction, Chemical carcinogenesis, Calcium signaling pathway, Gap junction, Protein digestion and absorption. However, the upregulated DEGs significantly enriched in pathways such as the IL-17 signaling pathway, NOD-like receptor signaling pathway, and Glutamatergic synapse, which differ from the downregulated DEGs involved in steroid hormone biosynthesis, retinol metabolism, and Renin secretion (Supplementary Fig. 1C-D).

**Fig. 3:**
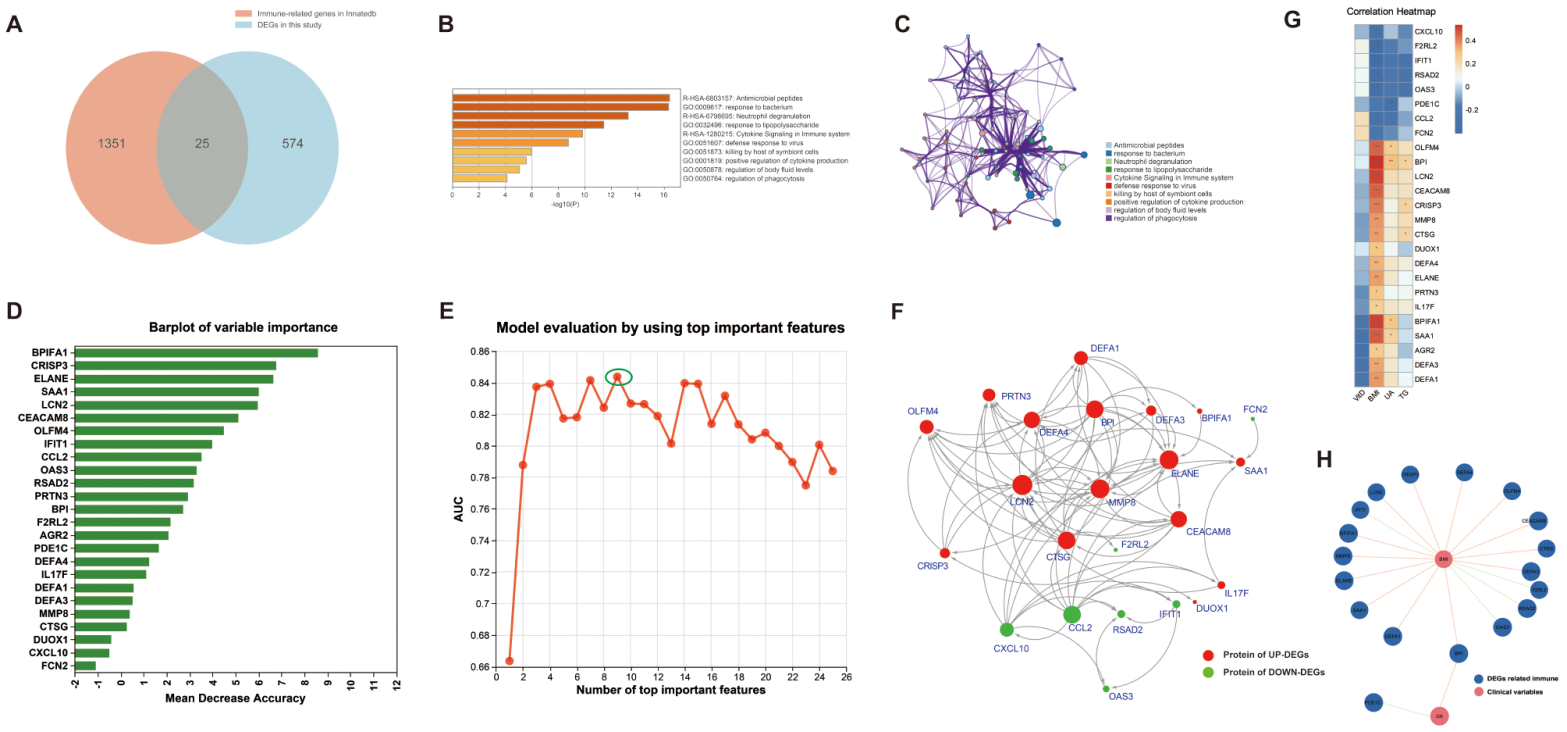
Functional analysis of immune-related DEGs and construction of the random forest model. (**A**) Venn diagram shows 27 genes that were common to DEGs and the InnateDB datasets. (**B**) Bar chart represents the enriched terms of immune-related DEGs (The parameter is set to P-value < 0.01, minimum = 3, and enrichment factor > 1.5). (**C**) Network diagram shows the enriched terms of immune-related DEGs. Each enrichment term is a node, nodes with the same color share the same cluster-ID. (**D**) The plot displays the importance ranking of 25 immune-related DEGs in the random forest model. The abscissa represents the importance value, and the ordinate represents the genes name. (**E**) AUC distribution plot of the random forest model. The random forest model achieved a better AUC of 0.844 by incorporating the top 9 ranked immune-related DEGs. (**F**) PPI network constructed based on immune-related DEGs. Red dots represent upregulated DEGs, while green dots represent downregulated genes. The larger size of the dots indicates more interactions with other genes in the network. (**G**) The heatmap shows the correlation between immune-related DEGs and clinical indices. Red indicates negative correlations, whereas blue indicates positive correlations. The squares with “*” represent | correlation coefficient | > 0.3 and *P*-value < 0.05. “*”: *P*-value < 0.05, “**”: *P*-value < 0.01, “***”: *P*-value < 0.001. (**H**) The network diagram shows the top 20 significant correlations between clinical variables and immune-related DEGs

The results of enrichment analysis indicated that DEGs participate in the pathological processes of obesity and its complications by maintaining or regulating the balance of the significantly enriched pathways mentioned above. Of course, the pathways and processes involved in upregulated and downregulated DEGs exhibited their characteristics. However, they are all involved in the signal pathways and biological functions related to immune and inflammatory responses, indicating that there was a close relationship between obesity and immunity. Then, we downloaded human immune-related genes cataloged in the InnateDB database (Supplementary Table 5) and compared them with DEGs to determine the gene intersection. Ultimately, we identified 25 immune-related DEGs (Fig. 3A) which are involved in pathways and processes such as antimicrobial peptides, neutrophil degranulation, positive regulation of cytokine production, etc. (Fig. 3B-C). After that, we constructed a random forest model to rank the importance of these genes (Fig. 3D). An AUC-validation method was employed to determine the optimal set of immune-related DEGs and the AUC for the model reaching its peak at nine genes (AUC = 0.844) (Fig. 3E), demonstrating a good discriminatory ability of the model. To explore the role of immune-related DEGs involved in childhood simple obesity, we built a PPI network. In this network, we noticed that the upregulated DEGs LCN2, ELANE, and MMP8 had the most numbers of nodes (Fig. 3F), all of which showed a significant positive correlation with BMI (correlation coefficient> 0.3, *P*-value < 0.05) (Fig. 3G). Additionally, the top 20 correlation (|correlation coefficient|>0.3, *P*-value < 0.05) between clinical variables and immune-related DEGs was illustrated in Fig. 3H. BPI, LCN2, BPIFA1, SAA1, and CEACAM8 were the top 5 genes highly correlated with BMI (with correlation coefficients of 0.54, 0.51, 0.51, 0.47, and 0.46, respectively). Notably, upregulated immune-related DEGs and partial downregulated including CCL2, CXCL10, IFIT1, OAS3, and RSAD2 are closely associated with IFN signaling. This indicates that these immune-related DEGs may play a regulatory role in the immune pathogenesis of obesity and its complications by modulating the balance of IFN signaling.

### Results of blood metabolomics profiling

**Fig. 4:**
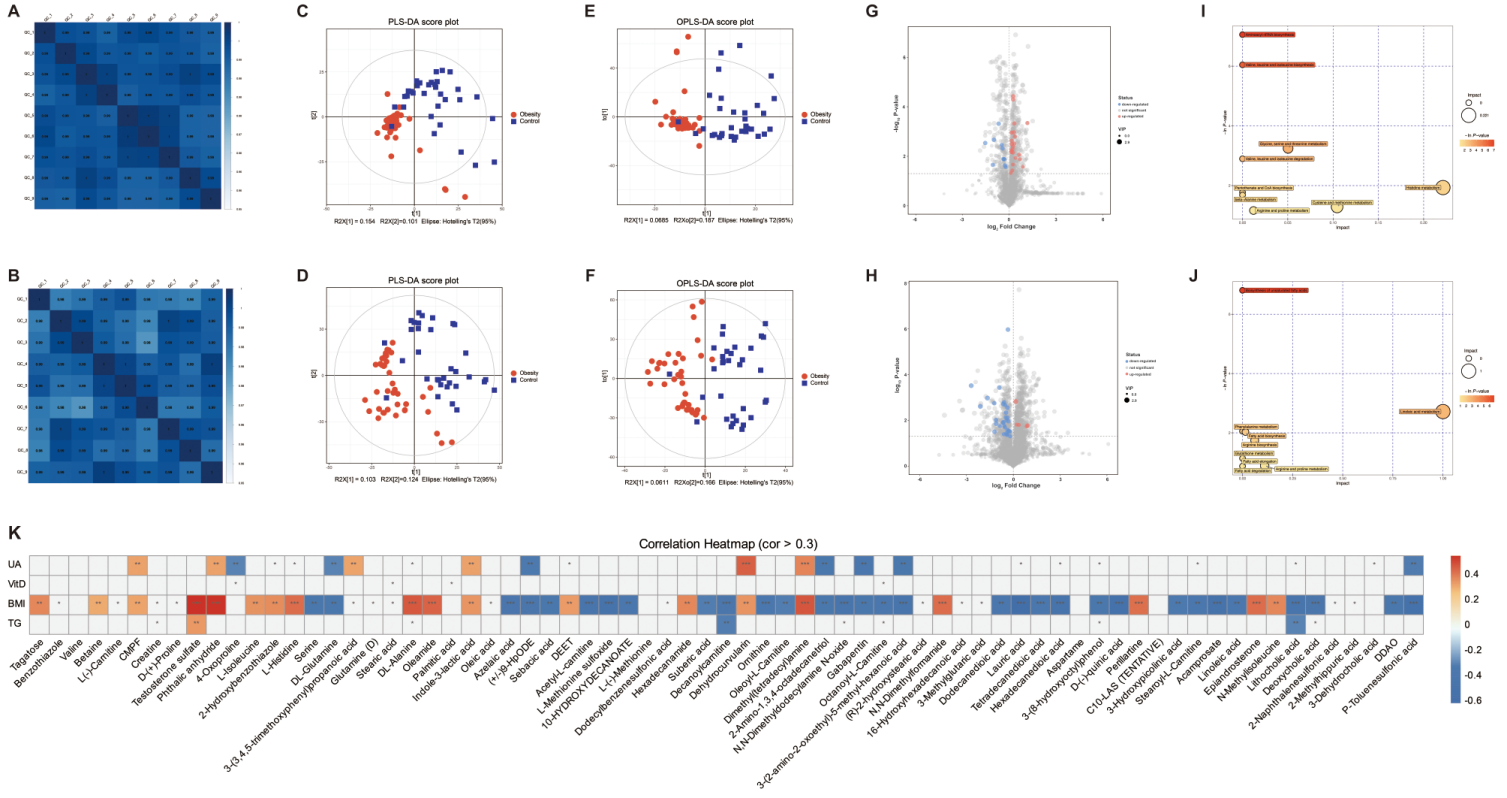
(**A**) and (**B**) represent the QC sample correlation analysis in positive and negative ion modes, respectively. The values in the squares represent the Pearson’s correlation coefficients. (**C**) and (**D**) show the clear segregation in plasma metabolites between the two groups based on the PLS-DA model under positive and negative ion modes respectively. (**E**) and (**F**) demonstrate the clear segregation in plasma metabolites between the two groups based on the OPLS-DA model under positive and negative ion modes respectively. (**G**) and (**H**) respectively show the volcano map of DEMs in positive and negative ion modes. Red represents upregulated DEMs, while blue represents downregulated DEMs. (**I**) and (**J**) are bubble diagrams of enriched pathways of DEMs under positive and negative ion modes, respectively. The abscissa indicates the impacts, and the ordinate represents the -ln of the *P*-value. The larger the bubble the greater the impact, the darker the color of the bubbler the greater the significance. (**K**) Heatmap of correlation between differentially identified metabolites and clinical variables. Red indicates a negative correlation whereas blue indicates a positive correlation. The squares with “*” represent | correlation coefficient | > 0.3 and *P*-value < 0.05. “*”: *P*-value < 0.05, “**”: *P*-value < 0.01, “***”: *P*-value < 0.001

In this study, we found that the Pearson correlation coefficients (R) between QC samples in both positive and negative ion modes were ≥ 0.98, indicating excellent assay stability and reproducibility, which ensure high-quality experimental data (Fig. 4A-B). Both Partial Least Squares Discriminant analysis (PLS-DA) and Orthogonal PLS-DA (OPLS-DA) analyses showed significant separation of samples between obesity and normal groups, indicating compositional differences in plasma metabolite between the two groups. At the same time, the samples within each group clustered together tightly reflecting good repeatability of intra-group samples (Fig. 4C-F). The experimental methods and results of this study demonstrated a high degree of accuracy and reliability.

Through data analysis, 71 significant differentially expressed metabolites were identified in the obesity group compared to the control group, with 27 upregulated and 44 downregulated. Among them, 34 were detected in positive ion mode and 37 in negative (Fig. 4G-H). These DEMs could be further classified into 17 subclasses (Table 1), and 5 metabolite subclasses accounted for the high proportions, with Amino acids and derivates (26.76%), Fatty acids and conjugates (21.13%), Amines (5.63%), Bile acids, alcohols and derivatives (4.23%), and Lineolic acids and derivatives (2.82%). These 17 subclasses were further categorized into 8 super classes, with Lipids and lipid-like molecules accounting for 42.25% and Organic acids and derivatives accounting for 29.58%.

**Table 1:** The classification of differentially expressed metabolites (DEMs)

Super Class	Class	Sub class	Metabolite	Formula	VIP	*P*-value	Relative content	Module
Orgaic nitrogen compounds	Organonitrogen compounds	Amines	2-Aminooctadecane-1,3,4-triol	C18H39NO3	1.8059	0.0048	DOWN	POS
Organic nitrogen compounds	Organonitrogen compounds	Amines	N, N-Dimethyldodecylamine N-oxide (DDNO)	C14H31NO	1.9517	0.0029	DOWN	POS
Organic nitrogen compounds	Organic nitrogen compounds	Amines	DDAO/((2-Methyl-6-[(2-methylpropyl)amino]heptan-2-OL))	C12H27NO	1.7595	0.0222	DOWN	POS
Organic nitrogen compounds	Organonitrogen compounds	Amines	dimethyl(tetradecyl)amine	C16H35N	1.6010	0.0067	UP	POS
Organic oxygen compounds	Organooxygen compounds	Alcohols and polyols	D-(-)-quinic acid	C7H12O6	2.4524	0.0003	DOWN	NEG
Organic oxygen compounds	Organooxygen compounds	Carbohydrates and carbohydrate conjugat	Tagatose	C6H12O6	2.4716	0.0015	UP	NEG
Organic nitrogen compounds	Organonitrogen compounds	Amino acids and derivatives	L(-)-Carnitine	C7H15NO3	1.5380	0.0031	UP	POS
Organic acids and derivatives	Carboxylic acids and derivatives	Amino acids and derivatives	DL-Glutamine	C5H10N2O3	1.3491	0.0131	DOWN	POS
Organic acids and derivatives	Carboxylic acids and derivatives	Amino acids and derivatives	Serine	C3H7NO3	1.1866	0.0273	DOWN	POS
Organic acids and derivatives	Carboxylic acids and derivatives	Amino acids and derivatives	Gabapentin	C9H17NO2	2.0396	0.0005	DOWN	POS
Organic acids and derivatives	Carboxylic acids and derivatives	Amino acids and derivatives	N-Methylisoleucine	C7H15NO2	1.6026	0.0011	UP	POS
Organic acids and derivatives	Carboxylic acids and derivatives	Amino acids and derivatives	Aspartame	C14H18N2O5	1.3078	0.0373	UP	POS
Organic acids and derivatives	Carboxylic acids and derivatives	Amino acids and derivatives	L-(-)-Methionine	C5H11NO2S	1.2491	0.0117	UP	POS
Organic acids and derivatives	Carboxylic acids and derivatives	Amino acids and derivatives	DL-Alanine	C3H7NO2	1.6604	0.0005	UP	POS
Organic acids and derivatives	Carboxylic acids and derivatives	Amino acids and derivatives	L-Histidine	C6H9N3O2	2.0728	0.0000	UP	POS
Organic acids and derivatives	Carboxylic acids and derivatives	Amino acids and derivatives	L-Isoleucine	C6H13NO2	1.7924	0.0011	UP	POS
Organic acids and derivatives	Carboxylic acids and derivatives	Amino acids and derivatives	Creatine	C4H9N3O2	1.6479	0.0071	UP	POS
Organic acids and derivatives	Carboxylic acids and derivatives	Amino acids and derivatives	D-(+)-Proline	C5H9NO2	1.5438	0.0086	UP	POS
Organic acids and derivatives	Carboxylic acids and derivatives	Amino acids and derivatives	Valine	C5H11NO2	1.1038	0.0216	UP	POS
Organic acids and derivatives	Carboxylic acids and derivatives	Amino acids and derivatives	Betaine	C5H11NO2	1.5420	0.0016	UP	POS
Organic acids and derivatives	Carboxylic acids and derivatives	Amino acids and derivatives	3-(2-amino-2-oxoethyl)-5-methyl-hexanoic acid	C9H17NO3	2.1067	0.0019	DOWN	NEG
Organic acids and derivatives	Carboxylic acids and derivatives	Amino acids and derivatives	4-Oxoproline	C5H7NO3	2.1054	0.0110	DOWN	NEG
Organic acids and derivatives	Carboxylic acids and derivatives	Amino acids and derivatives	D-Glutamine	C5H10N2O3	1.0338	0.0411	DOWN	NEG
Organic acids and derivatives	Carboxylic acids and derivatives	Amino acids and derivatives	Ornithine	C5H12N2O2	1.2901	0.0283	DOWN	NEG
Organic acids and derivatives	Carboxylic acids and derivatives	Amino acids and derivatives	L-Methionine sulfoxide	C5H11NO3S	1.2753	0.0130	DOWN	POS
Organic acids and derivatives	Carboxylic acids and derivatives	Carboxylic acids derivatives	N, N-Dimethylformamide	C3H7NO	1.6764	0.0041	UP	POS
Lipids and lipid-like molecules	Steroids and steroid derivatives	Bile acids, alcohols and derivatives	3-Dehydrocholic acid	C24H38O5	1.1635	0.0181	DOWN	NEG
Lipids and lipid-like molecules	Steroids and steroid derivatives	Bile acids, alcohols and derivatives	Deoxycholic acid	C24H40O4	1.9368	0.0317	DOWN	NEG
Lipids and lipid-like molecules	Steroids and steroid derivatives	Bile acids, alcohols and derivatives	Lithocholic acid	C24H40O3	2.6087	0.0011	DOWN	NEG
Lipids and lipid-like molecules	Steroid and steroid derivatives	Androstane steroids	Epiandrosterone	C19H30O2	1.7329	0.0032	UP	POS
Lipids and lipid-like molecules	Steroids and steroid derivatives	Sulfated steroids	Testosterone sulfate	C19H28O5S	1.8289	0.0171	UP	NEG
Lipids and lipid-like molecules	Fatty acyls	Fatty acids and conjugates	Acetyl-L-carnitine	C9H17NO4	1.9279	0.0021	DOWN	POS
Lipids and lipid-like molecules	Fatty acyls	Fatty acids and conjugates	Decanoylcarnitine	C17H33NO4	1.5285	0.0035	DOWN	POS
Lipids and lipid-like molecules	Fatty acyls	Fatty acids and conjugates	Octanoyl-L-Carnitine	C15H29NO4	1.3471	0.0135	DOWN	POS
Lipids and lipid-like molecules	Fatty acyls	Fatty acids and conjugates	Oleoyl-L-Carnitine	C25H47NO4	1.5340	0.0234	DOWN	POS
Lipids and lipid-like molecules	Fatty acyls	Fatty acids and conjugates	Stearoyl-L-Carnitine	C25H50NO4	1.3351	0.0127	DOWN	POS
Lipids and lipid-like molecules	Fatty acyls	Fatty acids and conjugates	(R)-2-hydroxystearic acid	C18H36O3	1.2069	0.0097	DOWN	NEG
Lipids and lipid-like molecules	Fatty acyls	Fatty acids and conjugates	16-Hydroxyhexadecanoic acid	C16H32O3	1.4796	0.0261	DOWN	NEG
Lipids and lipid-like molecules	Fatty acyls	Fatty acids and conjugates	3-Methylglutaric acid	C6H10O4	1.5390	0.0022	DOWN	NEG
Lipids and lipid-like molecules	Fatty acyls	Fatty acids and conjugates	Azelaic acid	C9H16O4	2.0360	0.0028	DOWN	NEG
Lipids and lipid-like molecules	Fatty acyls	Fatty acids and conjugates	CMPF/(3-Carboxy-4-methyl-5-propyl-2-furanpropionic acid)	C12H16O5	1.5260	0.0183	UP	NEG
Lipids and lipid-like molecules	Fatty acyls	Fatty acids and conjugates	Dodecanedioic acid	C12H22O4	1.4583	0.0174	DOWN	NEG
Lipids and lipid-like molecules	Fatty acyls	Fatty acids and conjugates	Hexadecanedioic acid	C16H30O4	2.0770	0.0004	DOWN	NEG
Lipids and lipid-like molecules	Fatty acyls	Fatty acids and conjugates	Lauric acid	C12H24O2	2.0877	0.0000	DOWN	NEG
Lipids and lipid-like molecules	Fatty acyls	Fatty acids and conjugates	Oleic acid	C18H34O2	1.9728	0.0047	DOWN	NEG
Lipids and lipid-like molecules	Fatty acyls	Fatty acids and conjugates	Palmitic acid	C16H32O2	1.8028	0.0078	DOWN	NEG
Lipids and lipid-like molecules	Fatty acyls	Fatty acids and conjugates	Sebacic acid	C10H18O4	1.4352	0.0313	DOWN	NEG
Lipids and lipid-like molecules	Fatty acyls	Fatty acids and conjugates	Stearic acid	C18H36O2	1.2482	0.0409	DOWN	NEG
Lipids and lipid-like molecules	Fatty acyls	Fatty acids and conjugates	Suberic acid	C8H14O4	1.4309	0.0498	DOWN	NEG
Lipids and lipid-like molecules	Fatty acyls	Fatty acids and conjugates	Tetradecanedioic acid	C14H26O4	1.5479	0.0315	DOWN	NEG
Lipids and lipid-like molecules	Fatty acyls	Fatty acids and conjugates	Hexadecanamide	C16H33NO	1.1353	0.0132	UP	POS
Lipids and lipid-like molecules	Fatty acyls	Fatty acids and conjugates	Oleamide	C18H35NO	1.4861	0.0005	UP	POS
Lipids and lipid-like molecules	Fatty acyls	Lineolic acids and derivatives	(+/-)9-HpODE	C18H32O4	1.2843	0.0202	DOWN	NEG
Lipids and lipid-like molecules	Fatty acyls	Lineolic acids and derivatives	Linoleic acid	C18H32O2	1.6026	0.0075	DOWN	NEG
Lipids and lipid-like molecules	Prenol lipids	Sesquiterpenoids	3-(8-hydroxyoctyl)phenol/(10betaH,11xi)-11-Hydroxy-13-nor-	C14H22O2	1.6986	0.0016	DOWN	NEG
Lipids and lipid-like molecules	Prenol lipids	Monoterpenoids	Perillartine	C10H15NO	1.5230	0.0053	UP	POS
Organic acids and derivatives	Hydroxy acids and derivatives	Medium-chain hydroxy acids and derivativ	10-hydroxydecanoate	C10H20O3	1.1661	0.0444	DOWN	NEG
Organic acids and derivatives	Organic sulfonic acids and deriv	Organosulfonic acids and derivatives	Acamprosate	C5H11NO4S	2.4766	0.0025	DOWN	NEG
Organic acids and derivatives	Pyridines and derivatives	Pyridinecarboxylic acids and derivatives	3-Hydroxypicolinic acid	C6H5NO3	1.6548	0.0375	DOWN	NEG
Organic acids and derivatives	Indoles and derivatives	Indolyl carboxylic acids and derivatives	Indole-3-lactic acid	C11H11NO3	1.2774	0.0156	UP	NEG
Other	Other	Other	Phthalic anhydride	C8H4O3	1.8930	0.0001	UP	POS
Other	Other	Other	Dodecylbenzenesulfonic acid	C18H30O3S	1.6693	0.0018	DOWN	NEG
Other	Other	Other	P-Toluenesulfonic acid	C7H8O3S	2.2182	0.0141	DOWN	NEG
Other	Other	Other	DEET/(N, N-Diethyl-2-methylbenzamide)	C12H17NO	1.5475	0.0060	UP	POS
Other	Other	Other	2-Methylhippuric acid	C10H11NO3	2.1515	0.0123	DOWN	NEG
Other	Other	Other	2-Naphthalenesulfonic acid	C10H8O3S	1.3562	0.0129	DOWN	NEG
Other	Other	Other	C10-LAS (TENTATIVE)	C16H26O3S	1.6189	0.0097	DOWN	NEG
Other	Other	Other	2-Hydroxybenzothiazole/2(3 H)-Benzothiazolone	C7H5NOS	1.6265	0.0023	UP	POS
Other	Other	Other	Benzothiazole	C7H5NS	1.0727	0.0471	UP	POS
Other	Other	Other	3-(3,4,5-trimethoxyphenyl)propanoic acid	C12H16O5	1.0071	0.0268	UP	POS
Other	Other	Other	dehydrocurvularin/5,6-Dihydro-11-methoxyyangonin	C16H18O5	1.2290	0.0161	UP	NEG

The alterations in metabolites could also influence corresponding metabolic pathways. Consequently, the KEGG pathway analysis on DEMs was conducted, identifying four pathways with significant dysregulation in childhood simple obesity (*P*-value < 0.05) (Table 2). The significantly changed pathways of DEMs in the positive ion mode between the two groups, were Aminoacyl-tRNA biosynthesis, Valine leucine and isoleucine biosynthesis, and Glycine, serine, and threonine metabolism (Fig. 4I). The DEMs, including L-Histidine, L-(-)-Methionine, Valine, L-Isoleucine, Betaine, and Creatine, which are enriched in the above pathways, presented higher abundance in obesity group (*P*-value < 0.05) (Table 3). Furthermore, the significantly altered pathway in the negative ion mode is the biosynthesis of unsaturated fatty acids (Fig. 4J), involving four DEMs (Palmitic acid, Stearic acid, Oleic acid, and Linoleate) mapped to this pathway, which exhibit lower abundance in obesity (Table 3). Moreover, the ROC curve of DEMs showed the TOP 4 AUCs were Lauric acid, Acamprosate, P-Toluensulfonic, and D-(-)-quinic acid, with 0.82, 0.78, 0.77, and 0.77, respectively. These findings suggested that Lauric acid had a strong ability as a disease biomarker in obese children (Supplementary Fig. 2). After consulting the literature, we found 12 plasma DEMs were associated with immunity, categorizing them into bile acid, short-chain fatty acid (SCFAs), hormone, tryptophan and its derivatives, and neurotransmitters (Table 4). Among these, the expression levels of Testosterone、Epiandrosterone、 L-histidine 、Indole-3-lactic acid, and CMPF were strongly positively correlated with BMI (correlation efficient *r* > 0.3, *P*-value < 0.05). The expression of Lithocholic acid、Deoxycholic acid、DL-Glutamine、Serine and 3-Hydroxypicolinic acid were strongly negatively correlated with BMI (correlation efficient *r* < -0.3, *P*-value < 0.05) (Fig. 4K).

**Table 2:** The enriched KEGG pathways of differentially expressed metabolites (DEMs). “POS”: positive, “NEG”: negative. “*”: *P*-value < 0.05, “**”: *P*-value < 0.01, “***”: *P*-value < 0.001

KEGG Pathway Description	Metabolite	*P*-value	Ion Module
Aminoacyl-tRNA biosynthesis	L-Histidine; L-(-)-Methionine; Valine; L-Isoleucine	0.0009***	POS
Valine, leucine and isoleucine biosynthesis	L-Isoleucine; Valine	0.0024**	POS
Glycine, serine and threonine metabolism	Betaine; Creatine	0.03883*	POS
Valine, leucine and isoleucine degradation	Valine; L-Isoleucine	0.05517	POS
Histidine metabolism	L-Histidine	0.1447	POS
Pantothenate and CoA biosynthesis	Valine	0.1696	POS
beta-Alanine metabolism	L-Histidine	0.1858	POS
Cysteine and methionine metabolism	L-(-)-Methionine	0.2770	POS
Arginine and proline metabolism	Creatine	0.3121	POS
Biosynthesis of unsaturated fatty acids	Palmitic acid; Stearic acid; Oleic acid; Linoleate	0.0011**	NEG
Linoleic acid metabolism	Linoleate	0.0660	NEG
Phenylalanine metabolism	2-Methylhippuric acid	0.1279	NEG
Fatty acid biosynthesis	Palmitic acid; Lauric acid	0.1313	NEG
Arginine biosynthesis	Ornithine	0.1745	NEG
Glutathione metabolism	Ornithine	0.3198	NEG
Arginine and proline metabolism	Ornithine	0.4083	NEG
Fatty acid elongation	Palmitic acid	0.4165	NEG
Fatty acid degradation	Palmitic acid	0.4165	NEG

**Table 3:** The differentially expressed metabolites (DEMs) in significantly enriched KEGG pathways. “POS”: positive, “NEG”: negative. “UP”: increased expression in the obesity group. “DOWN”: decreased expression in the obesity group

Metabolite	Expression levels	Ion module	Enriched KEGG Pathway
L-Methionine	UP	POS	Aminoacyl-tRNA biosynthesis
			Cysteine and methionine metabolism
L-Histidine	UP	POS	beta-Alanine metabolism
			Aminoacyl-tRNA biosynthesis
			Histidine metabolism
L-Valine	UP	POS	Aminoacyl-tRNA biosynthesis
			Valine, leucine and isoleucine biosynthesis
			Valine, leucine and isoleucine degradation
			Pantothenate and CoA biosynthesis
L-Isoleucine	UP	POS	Aminoacyl-tRNA biosynthesis
			Valine, leucine and isoleucine biosynthesis
			Valine, leucine and isoleucine degradation
Betaine	UP	POS	Valine, leucine and isoleucine degradation
Creatine	UP	POS	Valine, leucine and isoleucine degradation
Palmitic acid	DOWN	NEG	Biosynthesis of unsaturated fatty acids
			Fatty acid elongation
			Fatty acid degradation
			Fatty acid biosynthesis
Stearic acid	DOWN	NEG	Biosynthesis of unsaturated fatty acids
Oleic acid	DOWN	NEG	Biosynthesis of unsaturated fatty acids
Linoleic acid	DOWN	NEG	Biosynthesis of unsaturated fatty acids
			Linoleic acid metabolism
2-Methylhippuric acid	DOWN	NEG	Phenylalanine metabolism
Lauric acid	DOWN	NEG	Fatty acid biosynthesis
Ornithine	DOWN	NEG	Arginine biosynthesis
			Glutathione metabolism
			Arginine and proline metabolism

**Table 4:** The classification of immune-related differentially expressed metabolites (DEMs) based on literature review

Classification	DEMs
Bile acid	Deoxycholic acid
Bile acid	Lithocholic acid
Short-chain fatty acid	3-Methylglutaric acid
Hormone	Epiandrosterone
Hormone	Testosterone sulfate
Tryptophan and its derivativ	3-Hydroxypicolinic acid
Tryptophan and its derivativ	CMPF
Tryptophan and its derivativ	Indole-3-lactic acid
Neurotransmitter	DL-Glutamine
Neurotransmitter	Glutamine (D)
Neurotransmitter	L-Histidine
Neurotransmitter	Serine

Eventually, a total of 66 strong correlations (correlation efficient |r| > 0.3, *P*-value < 0.05) were identified through correlation analysis involving the 71 DEMs and clinical variables such as BMI, TG, Vit D, and UA using the Spearman method (Fig. 4K, and Supplementary Table 6). Among these correlations, the top 3 DEMs that exhibited significant correlations with BMI were Lauric acid (*r* = -0.62), Testosterone sulfate (*r* = 0.55), and Phthalic anhydride (*r* = 0.54). Additionally, only 3 DEMs significantly related to TG were Testosterone sulfate (*r* = 0.34), Decanoylcarnitine (*r* = -0.32), and Lithocholic acid (*r* = -0.30). Thirteen metabolites exhibited significant correlation with UA (correlation efficient |*r*| > 0.3, *P*-value < 0.05), The top 3 DEMs significantly correlated with UA were Dehydrocurvularin (*r* = 0.44), Dimethy(tetradecyl)amine (*r* = 0.38), and Gabapentin (*r* = -0.38).

### Results of 16 S rDNA sequence analysis

#### Diversity of the gut microbiota of childhood simple obesity

**Fig. 5:**
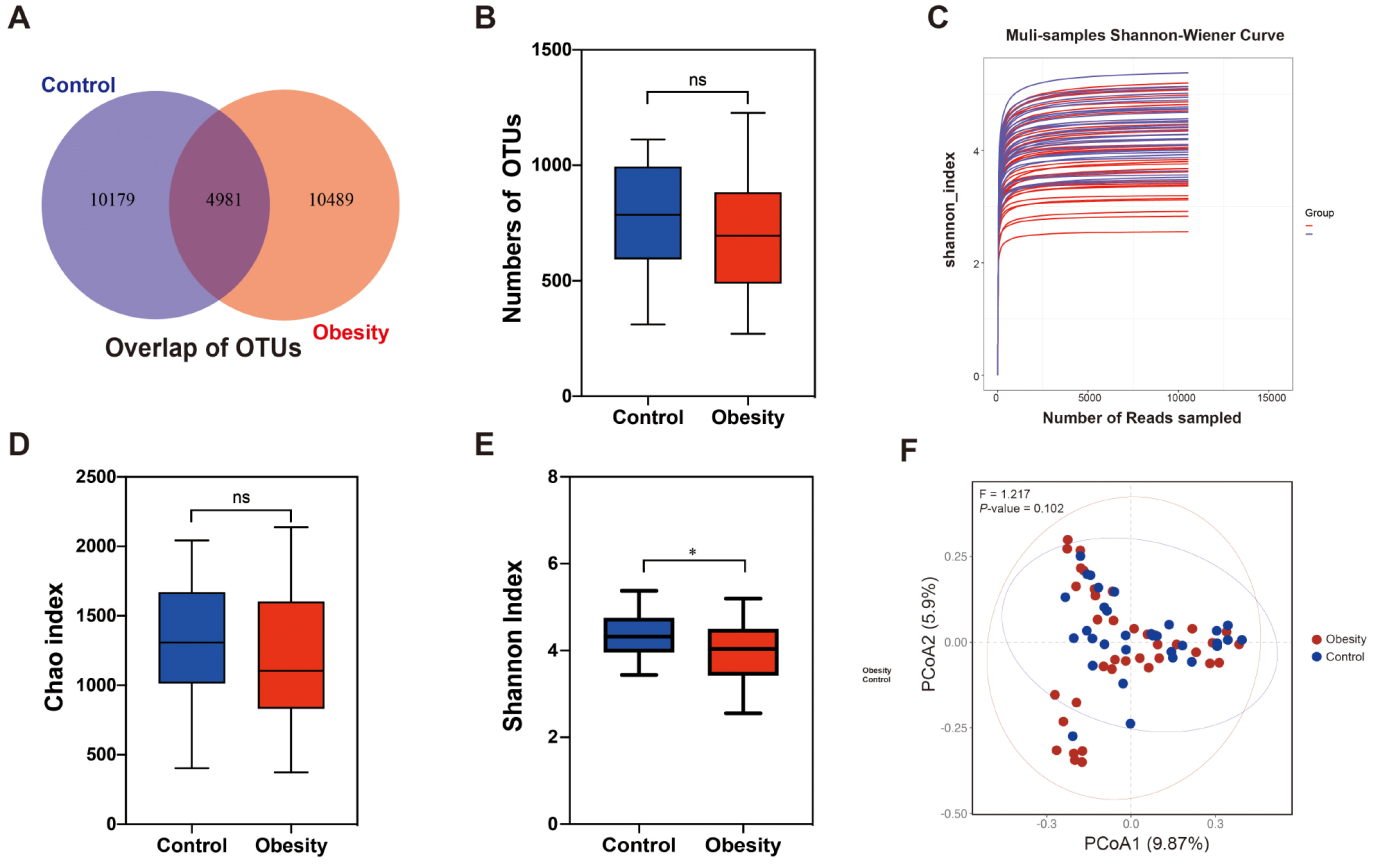
(**A**) Venn diagram showing the distribution of OTUs between obesity and normal control group, 4981 OTUs were shared in both groups. (**B**) The box plot shows no significant difference in OTU numbers between the two groups. (**C**) Shannon index curves of each sample in both groups tended to plateau as the sequencing number increased. (**D**) The diagram shows the *Chao* index of intestinal flora was no significant difference between the two groups. (**E**) The diagram exhibits the *Shannon* index of intestinal flora is significantly reduced in the obesity group, compared with the normal control group. (**F**) The beta diversity results are represented in the PCoA plot. “ns”: no significant, “*”: *P*-value < 0.05

After quality control, the 16 S rDNA sequencing data from 70 samples were included in the ultimate analysis. At the 97% similarity threshold, the number of Operational Taxonomic Units (OTUs) observed in both the normal control group and the obesity group was similarity, with 4981 shared OTUs identified between the two groups (Fig. 5A, Supplementary Table 7). Despite the lower overall count of OTUs in the obesity group compared to the normal group, this difference was not significant, possibly due to high inter-individual variability masking subtle differences between the two groups (Fig. 5B). The Shannon-Wiener curve between sequencing samples numbers and estimated richness, approached the saturation plateau trends in each sample (Fig. 5C), indicating that the data volume of sequenced samples was large enough to reflect the majority of microbial information in the samples. Alpha diversity analysis revealed that the Chao index was slightly lower in the obesity group than control group (*P*-value = 0.277) (Fig. 5D). However, the Shannon index of the obesity group was significantly lower than the normal control group (*P*-value = 0.034) (Fig. 5E), indicating lower species diversity in the obese children than in the normal children. Analysis of overall beta diversity using PCoA showed no significant difference between the two groups (Fig. 5F) (*F* = 1.217, *P*-value = 0.102, *P*-value evaluated via PERMANOVA) (*R* = 0.02, *P*-value = 0.137, *P*-value evaluated via ANOSIM). Notably, significant differences in beta diversity were observed between the obesity and normal control groups at the phylum, class, order, genus, and species levels, with the exception of the Family level (Supplementary Fig. 3). These results suggested that gut microbiota diversity was altered in the onset and development of obesity.

#### Alterations in gut microbiota composition

**Fig. 6:**
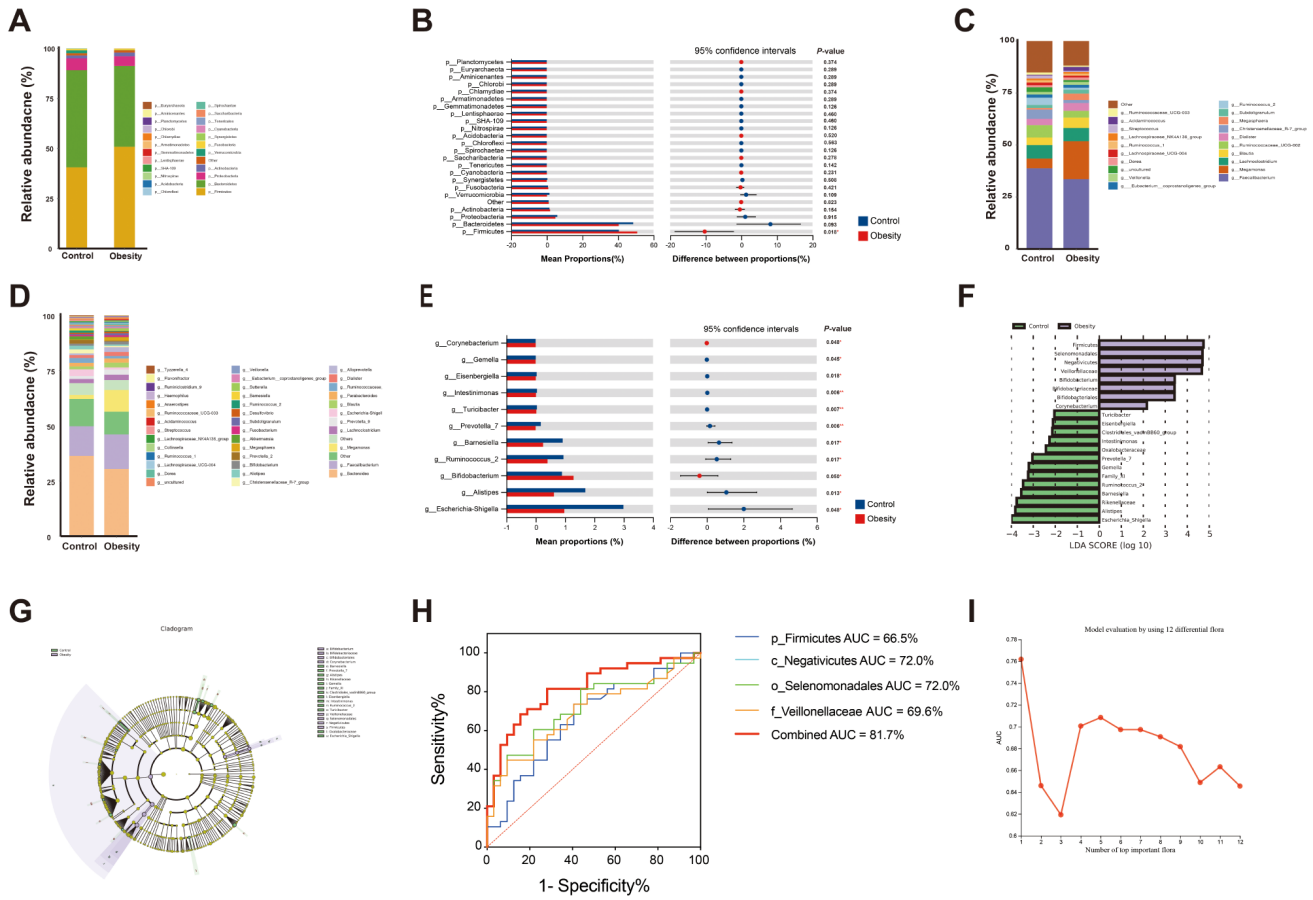
(**A**) and (**D**) Bar charts represent the relative abundance of bacterial communities in both groups at the phylum and genus levels, respectively. (**B**) and (**E**) Bar graphs show the difference in gut microflora between the two groups at the phylum and genus levels, respectively, with their 95% confidence intervals. The rightmost value represents the *P*-value. (**C**) The taxonomic bar chart shows the relative abundance proportion of bacterial genus within the *p_Firmicute* between the two groups. (**F**) Differential gut microflora selected by LEfSe analysis with |LDA score| >2 between the two groups. (**G**) The cladogram was obtained from the LEfSe analysis. The colored circles from inside to out represent the classification level (phylum, class, order, family, and genus). The diameter of each small circle represents their abundance. Yellow nodes represent species with no significant difference and differential gut microflora are colored according to the group. The control group was shown in green, and the obesity group in purple. (**H**) The corresponding AUC of differential gut microbiota axes of *p_Firmicutes*, *c_Negativicutes*, *o_Selenomonadales*, and *f_Veillonellaceae* identified in the LEfSe analysis. (**I**) AUC distribution plot of the random forest model based on the importance rank of the 12 differential gut flora at the phylum and genus. “*”: *P*-value < 0.05, “**”: *P*-value < 0.01

This study found 12 differential gut microbiotas (DGMs) between the two groups at the phylum and genus levels, with 1 phyla and 11 genera. At the phylum level, the gut microbiota compositions of the obesity and normal control groups were primarily comprised of *p_Firmicutes*, *p_Bacteroidetes*, *p_Proteobacteria*, and *p_Actinobacteria* for up to 97.91% and 96.49% of the relative abundance, respectively (Fig. 6A). It is noteworthy that, compared to the normal control group, the relative abundance of *p_Firmicutes* was significantly increased in the obesity group (*P*-value = 0.018) (Fig. 6B). The main genera responsible for the increase in *p_Firmicutes* abundance were identified as *g_Megamonas*, *g_Megasphaera*, *g_Blautia*, and *g_Acidaminococcus*(Fig. 6C). However, there were no significant differences in relative abundance between the two groups at other phyla levels (Fig. 6B). The *p_Firmicutes*/*p_Bacteroidetes* ratio was significantly higher in the obese group. Additionally, at the genus level, the four most predominant genera in the obesity group were *g_Bacteroids* (with 30.42%) proportions, *g_Faecalibacterium* (with 15.66%), *Other* (10.29%), and *g_Megamonas* (with 9.82%). However, in normal control group, the four most predominant genera were *g_Bacteroids* (with 30.42%), *g_Faecalibacterium* (with 15.66%), Other (with 12.3%), and *g_Escherichia-Shigella* (with 2.99%) (Fig. 6D). The relative abundance of most microbial at the genus level in the obesity group showed a decreasing trend, with significant decreases observed in *g_Intestinimonas*, *g_Prevotella7*, and *g_Turicibacter*, etc. (*P*-value < 0.05), while the relative abundance of *g_Bifidobacterium* was significantly increased (*P*-value < 0.05) (Fig. 6E).

Additionlly, LEfSe analysis also found that the phylum *p_Firmicutes* was predominant in the obesity group, while the genus *g_Escherichia Shigella* was in the normal control group (Fig. 6F). In evaluating the potential diagnostic and predictive value of differential gut microbiota identified in LEfSe analysis for obesity, we observed that the phylum *p_Firmicutes* with an AUC of 0.665 (Fig. 6G). However, when combining DGMs of *p_Firmicutes*, *c_Negativicutes*, *o_Selenomonadales*, and *f_Veillonellaceae* identified in the LEfSe analysis, the corresponding AUC reached 0.817 (Fig. 6H), exhibiting better performance compared to the random forest model based on differential gut microbiotas, which had an AUC of 76% (Fig. 6I). These findings suggested that the combined DGMs along this pathway had great potential for the diagnosis and prediction of obesity than individual DGMs.

#### Alterations in gut microbiota function

**Fig. 7:**
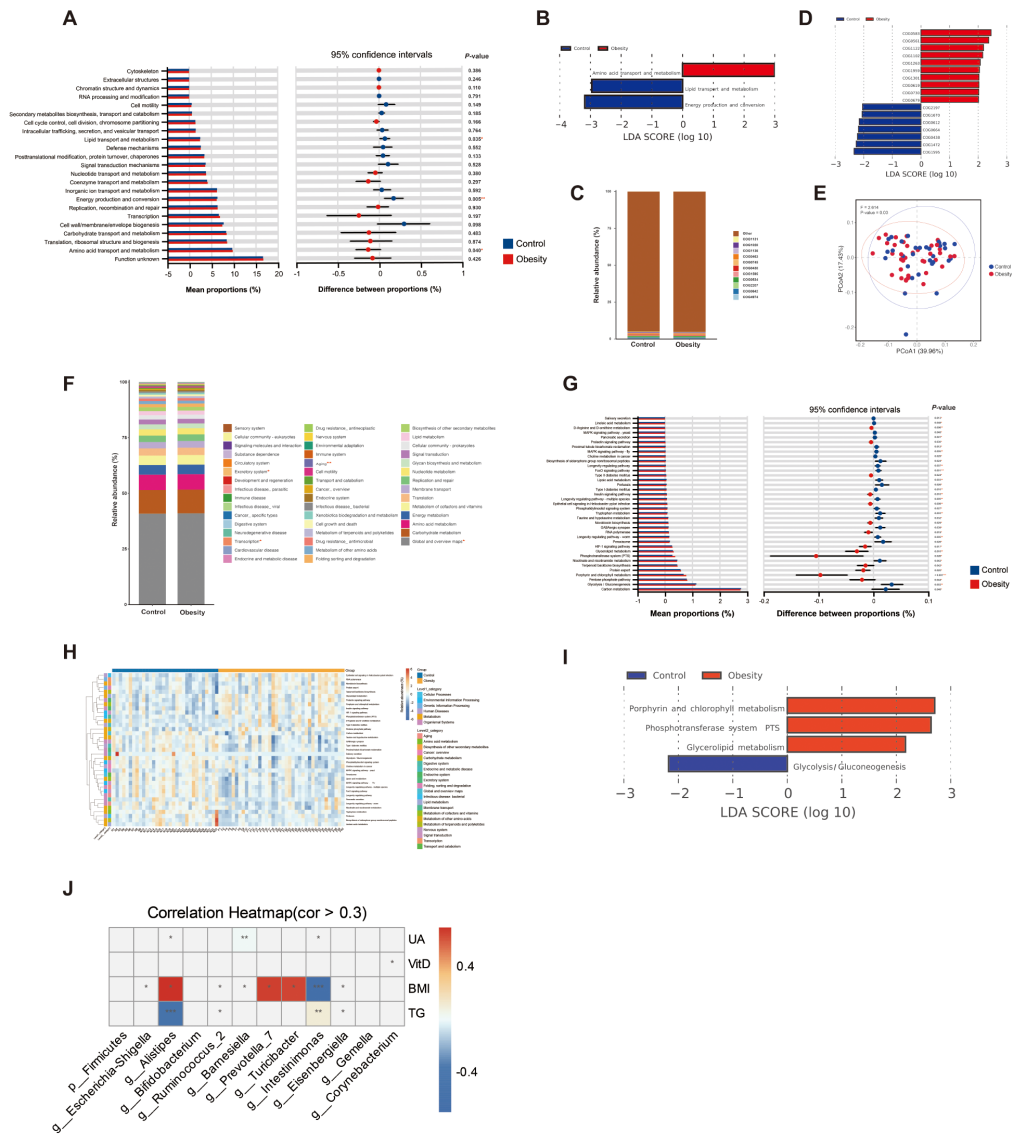
Changes in gut microbial function in obese child and correlation analysis between differential gut flora with clinical variables. (**A**) Analysis of the differences in the prediction of intestinal flora function annotation based on COG 1 level between the obesity and normal control group using the T-test, with their 95% confidence intervals. The rightmost value represents the *P*-value. (**B**), (**D**) and (**I**) respectively show differential function annotation based on COG 1 level, COG 2 level, and KEGG 3 level selected by Lefse analysis with |LDA score| >2 between the two groups. In the LDA score distribution histogram, the different colors represent their respective groups, and the length represents the LDA score, i.e., the degree of influence of pathways with a significant difference between the two groups. (**C**) COG level 2 functional relative abundance. (**E**) The PCoA analysis based on Bray-Curtis distance revealed significant separation between the obese and normal groups at the COG level 2. (**F**) Metabolic pathways at KEGG level 2 horizontal composition and comparison between the obesity and normal groups. The pathways marked with “*” were significant differences. (**G**) Analysis of the differences in the prediction of intestinal flora function annotation based on KEGG 3 level between the obesity and normal control group using the T-test, with their 95% confidence intervals. (**H**) The heatmap illustrates the distribution of 37 significantly different pathways at KEGG levels 1 and 2 within each sample. (**J**) Spearman correlation heatmap showing the correlation between differential gut microbiota and clinical variables. “*”: *P-* value < 0.05, “**”: *P-* value < 0.01, “***”: *P-* value < 0.001

To determine the potential impact of alterations in gut microbial composition on functional changes, we utilized 16 S rDNA sequencing data to predict biological functions through the application of PICRUST2 and pathway analysis. The results from Cluster of Ortholog Genes (COG) functional annotation showed that, at the COG level 1, the obesity group exhibited significantly increased relative abundance in the “Amino acid transport and metabolism” pathway (*P*-value < 0.05) compared to the control group, while the relative abundance enriched in “Energy production and conversion” and “Lipid transport and metabolism” was significantly decreased (*P*-value < 0.05) (Fig. 7A-B). At the COG level 2, the dominant categories for both groups were Site-specific recombinase XerD (COG4974) and Signal transduction histidine kinase (COG0642) (Fig. 7C). Lefse analysis revealed significant enrichment of COG0583 (DNA-binding transcriptional regulator, LysR family), COG0561 (Hydroxymethylpyrimidine pyrophosphatase and other HAD family phosphatases), COG1122 (Energy-coupling factor transporter ATP-binding protein EcfA2), etc. in the obesity group, while COG1595 (DNA-directed RNA polymerase specialized sigma subunit, sigma24 family), COG1472 (Periplasmic beta-glucosidase and related glycosidases), COG0438 (Glycosyltransferase involved in cell wall biosynthesis), COG0664 (cAMP-binding domain of CRP or a regulatory subunit of cAMP-dependent protein kinases), COG0612 (Predicted Zn-dependent peptidase), COG1670 (Protein N-acetyltransferase, RimJ/RimL family), and COG2197 (DNA-binding response regulator, NarL/FixJ family, contains REC and HTH domains) were significantly enriched in the normal group (Fig. 7D). Based on the Bray-Curtis distance of COG level 2 abundance, PCoA demonstrated a significant separation between the obesity and normal groups (Fig. 7E), suggesting there is variation in gut microbiome profiles between the two groups.

Furthermore, there were significant differences in the abundance of metabolic pathways between the two groups at KEGG level 2, namely the Global and overview maps, Aging, Transcription, and Excretory system (*P*-value < 0.05) (Fig. 7F). Moreover, we observed the abundances of 37 pathways were significantly different at KEGG level 3 as determined through the Wilcoxon rank sum test. (*P*-value < 0.05) )Fig. 7G). These pathways are not only associated with the metabolic pathways involved in the three major metabolites but also with the “Metabolism of cofactors and vitamins”, and “Metabolism of terpenoids and polyketides” (Fig. 7H). The LEfSe analysis revealed that the pathways of “Phosphotransferase system”, “Porphyrin and chlorophyll metabolism”, and “Glycerolipid metabolism” were significantly enriched in the obesity group (LDA > 2, *P*-value < 0.05), while Glycolysis/Gluconeogenesis played a significant role in the normal group (LDA > 2, *P*-value < 0.05) (Fig. 7I).

#### Construction of multi-omics correlation network

**Fig. 8:**
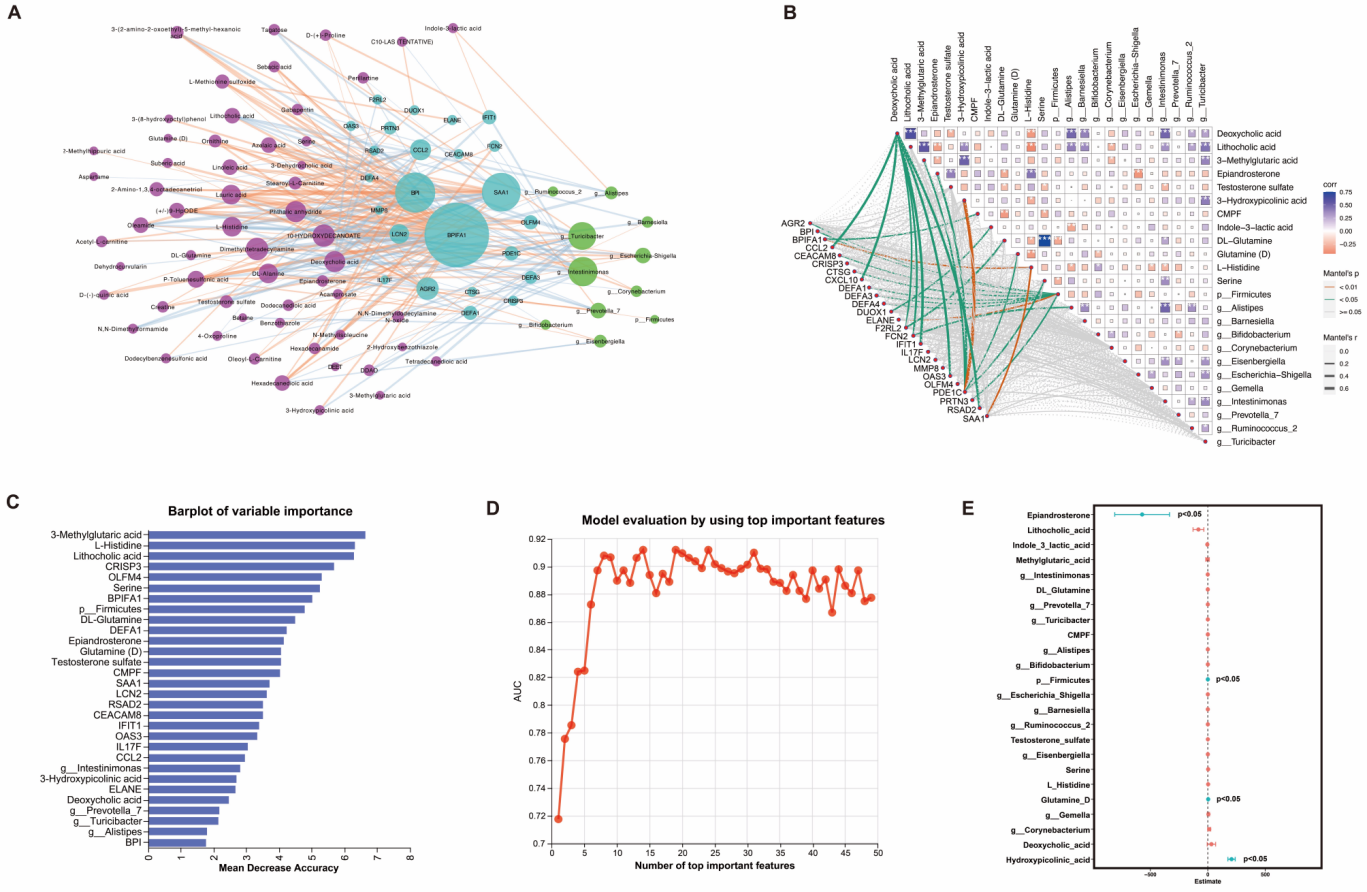
Integrative multi-omics network based on Spearman correlation and constructing a multivariable linear regression model. (**A**) Correlation Network among immune-related DEGs, immune-related DEMs, and differential gut microbiota. Only correlations associated with the absolute value of Spearman correlation coefficient > 0.3 are presented in the network. The thicker the line, the stronger the correlation. (**B**) Correlation heatmap, among immune-related DEGs, immune-related immune-related DEMs, and differential gut microbiota, based on the mantel test. The upper right triangle represents the relationship between immune-related differential metabolites and differential gut microbiota (a total of 24 variables). The color gradient indicates the Spearman correlation coefficient. Red and blue denote negative and positive correlations, respectively. Darker colors or larger rectangle areas indicate higher absolute correlation coefficients. Asterisks denote the significance of the correlation. The connecting lines in the middle represent the relationship between immune-related differential genes and the other 24 variables. Line color indicates the range of *P*-value, solid lines represent positive correlation coefficients, and line width indicates the magnitude of Mantel’s r. (**C**) The plot displays the importance ranking of 49 variables in the random forest model of obesity, including 25 immune-related DEGs, 12 immune-related DEMs, and 12 differential gut flora. The abscissa represents the importance value, and the ordinate represents the variable name. (**D**) AUC distribution plot of the random forest model. The random forest model incorporates the top-ranking 14 variables with the best AUC of 0.912. (**E**) The visualization of the constructed multiple linear regression model

To explore the inter-omics correlation, we performed Spearman analysis on the 25 immune-related DEGs, 71 DEMs, and 12 DGMs. Then we visualized all significant inter-omics associations with a correlation coefficient above 0.3 or below − 0.3 (*P*-value < 0.05) (Supplementary Tables 8 and Fig. 8A). A total of 223 significant correlations were identified in the integrative multi-omics network, with immune-related DEGs BPIFA1, BPI, and SAA1 accounting for a large proportion of interactions. Furthermore, DEMs, such as Dimethy (tetradecyl) amine, 10-HYDROXYDECANOATE, Deoxycholic acid, Phthalic anhydride, and DL-Alanine, also exhibited better connections in the network. Additionally, DGMs of the genus *g_Intestinimonas* and *g_Turicibacter* showed the strength of the interactions in the network.

The products released by gut microbiota, such as antigens and metabolites, could influence the expression of immune-related genes, thereby modulating the host immune system. To further explore the potential regulation of differential gut microbiota and DEMs on immune-related DEGs, we applied the Mantel test method to investigate the relationships among 25 immune-related DEGs, 12 immune-related DEMs, and 12 DGMs. Notably, the results revealed that PDE1C, Deoxycholic acid, and *p_Firmicutes* exhibited numerous significant interactions (Fig. 8B). Simultaneously, we constructed a random forest model to rank the 49 differential molecules, and the model achieved its highest AUC of 0.912 when including 14 molecules (Fig. 8C-D), demonstrating the superior discriminating performance for childhood obesity. The 14 molecules corresponding to the model at this peak AUC value included 3-methylglutaric acid, L-histidine, Lithocholic acid, CRISP3, OLFM4, *p_Firmicutes*, DL-Glutamine, DEFA1, Epiandrosterone, D-Glutamine, Testosterone sulfate, and CMPF, which could serve as a biomarker set.

Subsequently, a multiple linear regression analysis was conducted using immune-related DEGs as the dependent variable and DEMs and DGMs as independent variables. Eventually, we created a multivariable linear regression model, expressed by the combination of each variable and the corresponding coefficient, namely PDE1C = -0.004* *p_Firmicutes* + 3.059*DL-Glutamine − 571.6*Epiandrosterone + 206.5*3-Hydroxypicolinic-acid (Fig. 8E).This model suggested that the plasma metabolites Epiandrosterone, 3-Hydroxypicolinic_acid, Glutamine_D, and the gut microbiota *p_Firmicutes* may synergistically impact the expression levels of the PDE1C gene in the blood of obese children, promoting the onset and development of obesity and its associated complications.

## Discussion

This study investigated the transcriptomics, metabolomics, and gut microbiota characteristics of childhood simple obesity and identified novel biomarkers associated with immunity. Previous studies have shown a close association between obesity and immunity. Local vascular supply became restricted, caused by adipocyte hypertrophy and hyperplasia in the obese states could result in cell stress or cell death, leading to the subsequent release of disease-associated molecular patterns (DAMPs) into the extracellular environment, and ultimately triggering infiltration and activation of immune cells, such as proinflammatory M1 macrophages. Adipocytes also contribute to the development of obesity-induced inflammation by increasing the secretion of MCP-1, TNF-α, and IL-6. Transcriptome sequencing in this study revealed that DEGs were mapped to many immune-related signaling pathways, such as the IL-17 signaling pathway, chemokine activity, and cytokine-cytokine receptor interaction. IL-17 A, a dominant member of the IL-17 family, played a crucial role in promoting obesity and inducing metabolic disorders, and aberrant autoimmune responses associated with obesity [22, 23]. Interleukin-17 F (IL-17 F) frequently forms dimers with interleukin-17 A (IL-17 A) and initiates downstream signaling via the IL-17RA and IL-17RC receptor complex. Both cytokines are upregulated in a variety of inflammatory tissues and synergistically enhance the inflammatory response in conjunction with other pro-inflammatory mediators, such as tumor necrosis factor (TNF) [24]. Evidence from a randomized controlled clinical trial indicates that IL-17 F is a critical contributor to chronic tissue inflammation [25]. Furthermore, IL-17 F expression levels exhibit a positive correlation with body mass index (BMI) and increase in proportion to the amount of subcutaneous fat in the limbs. There is also a positive correlation between IL-17 F levels and the incidence of atherosclerosis [26]. Our study found that the expression of the IL-17 F gene was increased in the obesity group and was significantly positively correlated with BMI. These findings suggested that targeting the IL-17 F signaling pathway might be an effective strategy against obesity and its complications. Identified upregulated and downregulated DEGs were enriched in the IL-17 signaling pathway. Therefore, further investigation of the complex role of this pathway in childhood simple obesity is essential.

KEGG analysis showed some DEGs had significant enrichment in neuroactive ligand-receptor interaction, including CHRM5, GRID1, F2RL2, PPY, HTR2B, CTSG, GRM5, GRIK4, and OPRD1. While most of these are strongly associated with various neuropsychiatric disorders such as sleep modulation, stress response, anxiety, bipolar disorder, impulsive behavior, impaired social skills, Alzheimer’s disease, and schizophrenia [27–32]. There is a causal relationship between obesity and brain functional abnormalities. This is evident not only in the transition from obesity to neuropsychiatric disorders but also in endothelial dysfunction and vascular inflammation caused by obesity, which accelerates neuronal loss within brain regions [33]. In addition, obese individuals typically demonstrate poorer performance in memory, attention, verbal fluency, and executive functions, which could be explained by the alterations in the brain caused by obesity [33]. This study also revealed that changes in these DEGs in obese individuals may potentially affect brain function through glutamate signaling, proenkephalin, and serotonin, ultimately leading to the onset of neuropsychiatric disorders.

This study identified 25 immune-related DEGs, some of which were known to be associated with obesity, such as ELANE, SAA1, LCN2, OLFM4, CCL2, OAS3, BPI, AGR2, IL-17 A, DEFA1, DEFA3, MMP8, CTSG, DUOX1, and CXCL10, while BPIFA1, CRISP3, CEACAM8, IFIT1, RASD2, PRTN3, F2RL2, PDE1C, DEFA4, and FCN2 have not been reported to be related to obesity. In the PPI network constructed based on these genes, LCN2, ELANE, and MMP8 processed the most nodes, indicating their key roles in the obesity-related immune network. The lipocalin-2 (LCN2) protein, encoded by the LCN2 gene, is a pleiotropic inflammatory cytokine highly expressed in adipose tissue. LCN2 participated in obesity and its metabolic complications such as T2DM, and cardiovascular diseases, which may relate to the activation of LCN2 signaling (such as TNF-α/NLRP3/LCN2) inducing mitochondrial dysfunction, oxidative stress, insulin resistance, and macrophage activation in adipocytes [34–36]. Knockout of the ELANE gene in mice lead to an increase in circulating leptin levels, which increases fatty acid oxidation in the liver and brown adipose tissue and uncoupling protein 1 expression in brown adipose tissue. These changes eventually lead to ELANE knockout mice displaying resistance to high-diet-induced weight gain, insulin resistance, inflammation, and fatty liver [37]. Overweight and fat accumulation are associated with elevated circulating levels of MMP-8 [38, 39]. The increased MMP-8 may promote weight gain and insulin resistance in obese individuals by cleaving and degrading the human insulin receptor, making obese patients more susceptible to atherosclerosis and increasing long-term mortality [39, 40]. This upregulation of MMP-8 by Neuropeptide Y facilitates macrophage migration [41].

In this study, the BPIFA1 gene is increased in the obesity group. The protein encoded by the BPIFA1 gene is a lipid-binding protein mainly expressed in the respiratory tract, with antimicrobial, immune regulatory, and regulating smooth muscle contraction functions [42]. BPIFA1 is identified as a modifier gene for asthma and its overexpression in non-infectious mouse models increases systemic inflammation [43, 44]. Given the higher risk of asthma, more severe symptoms, poorer symptom control, and more frequent exacerbations in obese children [44], we suspect BPIFA1 could play a certain role in this. The CRISP3 protein belongs to the cysteine-rich secretory protein family [45], existing in neutrophils and eosinophils in either glycosylated or non-glycosylated forms [46]. Current research on the CRISP3 gene is mainly focused on tumors, and its overexpression was involved in the development of prostate cancer, lung cancer, and breast cancer [47–49]. Therefore, the increased CRISP3 could promote tumor generation in obese individuals. The protein encoded by the CEACAM8 gene is a glycoprotein with immune-regulatory functions [50], expressed only in human neutrophils [51], and is considered a marker for neutrophil activation. The secretion of soluble CEACAM8 could trigger an excessive immune response, especially in autoimmune diseases such as rheumatoid arthritis [52]. However, obesity could promote breast cancer cells metastasis to the lungs in a neutrophil-dependent manner [53]. In female gastric adenocarcinoma, the abundance of CEACAM8-positive tumor-infiltrating neutrophils had a specific impact on the prognosis [54]. The protein encoded by the PRTN3 gene is one of the main components of neutrophils and is involved in the activation and processing of pro-inflammatory cytokines associated with atherosclerosis, such as IL-1β, TNF-α, and MCP-1 [55]. By inhibiting the expression of membrane-associated protein A1, PRTN3 eventually promoted atherosclerosis-related inflammatory responses. The enzyme encoded by the PDE1C gene is highly expressed in cardiac muscle and regulated the proliferation, migration, and intimal hyperplasia of vascular smooth muscle cells, and pathological vascular remodeling [56]. PDE1C inhibitors promoted vasodilation and blood pressure lowering [57]. Moreover, studies have found that increased PDE1C protein levels promoted cognitive impairment possibly via reducing the cAMP levels in the hippocampus [58]. The defensin, encoded by the DEFA4 gene, is an antimicrobial and cytotoxic peptide mainly found in neutrophils and recruits dendritic cells and T cells to the sites of bacterial invasion. Furthermore, DEFA4 exhibits pro-inflammatory activity in the intestine by activating macrophages and amplifying local inflammatory responses, leading to intestinal permeability and systemic inflammation [59], which plays a role in autoimmune diseases such as inflammatory bowel disease. The high expression of the above-described immune-related DEGs recognized in this study could promote obesity and its complications.

As an interferon-stimulated gene, IFIT1 responded to interferon (IFN) signals and had anti-inflammatory and anti-apoptosis. It was reported that IFIT1 could reduce the expression of TNF-α, IL-1β, and IL-6 in macrophages of mice and alleviate apoptosis in mouse lung epithelial cells induced by macrophage supernatant [60]. The protein encoded by the RASD2 gene is a small GTPase protein, highly expressed in the striatum of the brain and regulated mental, cognitive, and motor functions [61–63]. Additionally, RASD2 could protect neurons by removing damaged mitochondria through mitophagy [64], playing an important role in diseases such as Alzheimer’s disease. The protein encoded by the F2RL2 gene is protease-activated receptor 3 (PAR3), a G protein-coupled receptor. Thrombin stimulates insulin secretion through PAR3 and blocking the cleavage of PAR3 was expected to counteract insulin resistance and β-cell failure driven by excessive insulin secretion in T2DM [65]. The FCN2 was highly expressed by hepatocytes and abundantly secreted into the circulation. It has been found that plasma FCN2 expression was decreased in patients with pathological obesity and negatively correlated with the stage of liver fibrosis in patients with non-alcoholic fatty liver disease [66]. Compared to obesity itself, these immune-related DEGs play more roles in obesity-related complications. The changes in these genes after obesity increase the risk of obesity-related complications. Interestingly, these DEGs are closely related to INF signals and neutrophil activation. Further research into the roles of these genes in the pathological processes of obesity and its complications, is expected to reveal more refined and complex pathological features of obesity.

The metabolomic analysis in this study revealed significant alterations in plasma metabolite levels in obese patients, characterized predominantly by elevated levels of lipids and lipid-like molecules, followed by organic acids and their derivatives. Among them, well-known obesity-related metabolites such as isoleucine, valine, glutamine, and methionine were significantly increased in the obesity group. Additionally, betaine and creatine were significantly increased in the obesity group. Betaine, a major methyl donor, induced browning of inguinal white adipose tissue (iWAT) and activation of brown adipose tissue (BAT) by modulating the miR-378a/YY1 axis, thereby improving obesity and its complications [67]. Creatine plays a crucial role in the function of immune cells. For example, creatine kinase B regulates T-cell proliferation and activation by modulating T-cell receptor signaling and mediates macrophage polarization and phagocytic function by inhibiting the IFN-γ/JAK/STAT/iNOS axis and promoting the IL-4/STAT6/ARG1 axis [68]. Furthermore, the selective creatine depletion of adipocytes results in decreased whole-body energy expenditure [69]. These studies suggest that investigating the immune-regulatory mechanisms of creatine in obesity may contribute to the development of therapies targeting specific metabolic requirements.

Additionally, this study also found significant decreases in certain metabolites, including oleic acid (OA) and lauric acid (LA). As we all know, unsaturated fatty acids are beneficial to human health. OA, the most abundant monounsaturated fatty acid, and its derived oleoylethanolamide (OEA) could enhance lipid utilization by triggering downstream signaling cascades peroxisome proliferator-activated receptor α (PPAR-α) and reduce food intake by inducing a sense of satiety [70–73]. Importantly, OA reduced the secretion of IL-6 and MCP-1 in adipose tissue, exhibiting anti-inflammatory effects. Furthermore, OA rescued palmitic acid-induced T-cell depletion and then improved T-cell-mediated antitumor immunity in obese mouse models. LA reduced plasma total cholesterol levels, insulin resistance, and the risk of cardiovascular disease while improving cognitive function in Alzheimer’s disease patients [74–76]. By promoting whole-body energy metabolism, LA supplementation decreased HFD-induced exaggerated body weight and fat content in mice, thereby improving obesity [77]. Therefore, based on these findings, we think supplementation of these substances will be likely to improve obesity and provide new treatment methods.

This study identified 12 DEMs, 2 were affiliated with bile acids, 1 was affiliated with short-chain fatty acids, 2 were affiliated with s hormones, 3 were affiliated with tryptophan and its derivatives, and 4 were affiliated with neurotransmitters (Table 4), could be directly and indirectly participate in the immune response. In the following section, we described in detail their potential impacts on the occurrence and development of obesity. As secondary bile acids, deoxycholic acid and lithocholic acid are secondary bile acids that regulate lipid levels and promote obesity by affecting cholesterol metabolism. Deoxycholic acid and lithocholic acid are also the most potent agonists of transmembrane G protein-coupled bile acid receptor 5 (TGR5) [78]. In brown adipose tissue and muscle, activation of TGR5 converts thyroxine to triiodothyronine promoting energy expenditure and reducing obesity [79]. Moreover, TGR5 regulates mucosal immune homeostasis and deficiency of TGR5 in macrophages could induce inflammation and diet-induced obesity [80]. Therefore, decreased expression of deoxycholic acid and lithocholic acid in obese states increased circulating cholesterol and lipids levels, and also weakened the anti-obesity function of TGR5, ultimately promoting obesity. Hormones play an important role in obesity and its metabolic complications. This study found that the expression of two steroid hormones, epiandrosterone, decreased in obese children, while the expression of testosterone sulfate increased, reflecting abnormal metabolism of androgens/testosterone in obese children. It has been reported that decreased testosterone levels caused by obesity resulted in impaired testicular function and male reproductive dysfunction. Lack of androgens further accelerates adipose tissue expansion, exacerbating obesity and aggravating gonadal dysfunction, which is a vicious cycle [81]. The role of tryptophan and its derivatives in obesity has been seldom studied. As an iron chelator, 3-Hydroxypicolinic acid reduces circulating iron accumulation by binding to Fe^3+^ [82]. This study found decreased expression of 3-hydroxypicolinic acid in the obesity group, which may lead to iron overload in circulation, causing multi-organ damage. Elevated plasma CMPF expression was observed in the obesity group. Previous studies have shown increased expression of CMPF in the serum of patients with chronic heart failure and mice, and increased CMPF damages the cardiac function and exacerbates myocardial injury by enhancing fatty acid oxidation [83]. Moreover, CMPF induced oxidative stress responses by directly damaging β-cell mitochondria, reducing insulin biosynthesis, and impairing glucose tolerance [84]. More importantly, CMPF is closely related to vitamin D (Vit D), and higher levels of circulating CMPF may inhibit the hydroxylation and metabolism of 25(OH)D [85], reducing the levels of active 1,25(OH)_2_D. 1,25(OH)_2_D plays a crucial role in maintaining immune system balance by regulating the function of various immune cells and suppressing inflammatory responses through multiple pathways. In this study, we also found the expression of active Vit D was decreased in obesity. Therefore, research on CMPF may help to elucidate the possible mechanisms for decreased Vit D and immune imbalance in obese children. Indole-3-lactic acid (ILA) is an indole compound derived from the gut microbiota involved in tryptophan metabolism, which regulates immune responses through the aryl hydrocarbon receptor and participates in anti-inflammatory and allergic responses [86–88]. Furthermore, ILA inhibites lipid accumulation in vitro and has the potential to counteract obesity [89]. However, in our study, ILA expression is increased in the obesity group. From this perspective, we understand that in the obese state, the body’s metabolism appears to be in a dynamic self-regulating process to maintain health as much as possible.

This study found that in the obesity group, the expression of DL-Glutamine, Glutamine (D), and Serine decreased, while L-Histidine increased. These metabolites not only play important roles in whole-body central metabolism but also serve as crucial neurotransmitters or their precursors in the human body. Glutamine, one of the most abundant amino acids in the body, acts as both an energy substrate for the tricarboxylic acid cycle and a nitrogen donor, participating in various metabolic processes [90]. Petrus et al. discovered decreased levels of glutamine in obese individuals [91], while supplementation of glutamine could alleviate body weight and fat mass, and improve insulin sensitivity, and glucose homeostasis [92–94]. Furthermore, changes in glutamine metabolism could also regulate pro-inflammatory pathways in white adipose tissue (WAT). Specifically, glutamine directly linked obesity to inflammation. When glutamine levels decrease in adipocytes, it increases the levels of UDP-N-acetylglucosamine and O-GlcNAcylation of chromatin-binding proteins near inflammatory genes, thereby activating pro-inflammatory factors such as IL-1β and IL-6 [91]. Supplementation with glutamine could reduce the expression of pro-inflammatory genes and proteins in adipocytes, decrease macrophage infiltration in WAT, and exert anti-inflammatory effects [91]. Glutamine could also inhibit allergic airway inflammation by upregulating MAPK phosphatase 1 [95]. Overall, glutamine metabolism plays a crucial role in regulating inflammatory immune responses. Serine transmits signals through synapses between neurons. The potential role of serine in obesity has been detailed in the previous section. Histidine possesses beneficial properties such as antioxidant, anti-inflammatory, anti-glycation, and chelating activities, and is associated with obesity and chronic heart failure [96]. Histidine can promote the transport of free fatty acids into mitochondria for oxidation, improving obesity. This is specifically reflected in the sustained down-regulation of de novo lipogenesis genes and TG accumulation in nonalcoholic fatty liver disease (NAFLD) animal models and leptin-deficient mice after histidine treatment [97]. Supplementation with histidine can also improve insulin resistance and diabetes in obese individuals by downregulating inflammatory responses [98]. Additionally, histamine generated from histidine acts as a neurotransmitter in the brain, participating in anxiety, stress responses, learning, and memory. Histidine can also be converted to uric acid, which, upon crossing the blood-brain barrier, promotes glutamate biosynthesis and its release in various brain regions, thereby enhancing learning and memory abilities [99]. Consequently, a decrease in serum histidine levels may impair brain functions.

The gut microbiota has emerged as a crucial factor in regulating host physiology and metabolism [100]. It has been established that the gut microbial community is associated with obesity and inflammation. Recent research indicated that dysbiosis of obesity-associated microbiota is sufficient to promote weight gain, inflammatory response, and proliferation of immune cells in the adipose tissue, independent of genetics and dietary manipulation [101]. Our study found that compared to the normal control group, the gut microbial richness was reduced in the obesity group, with an increase in the ratio of *p_Firmicutes*/*p_Bacteroidetes*, which is consistent with previous findings [102]. Previous studies have shown that overweight adolescents exhibit increased abundance of *g_Bacteroides* and *g_Bifidobacterium* genera, while the presence of *g_Prevotella* is decreased. However, the opposite was observed with weight loss and maintenance participants [103]. *g_Prevotella* is known for its high fiber utilization capability and high total short-chain fatty acid secretion, promoting healthy dietary patterns and aiding in weight loss [104]. As common probiotics, *g_Bifidobacterium* plays a significant role in inhibiting harmful bacteria growth, enhancing intestinal mucosal immune function, and reducing the risk of intestinal inflammation. This study also observed a significant decrease in *g_Prevotella* _7 in obese patients, and negatively correlated with immune-related DEGs OLFM4 and CRISP3. While *g_Bifidobacterium* showed a significant increase and positively correlated with LCN2. Both *g_Prevotella_7* and *g_Bifidobacterium* are potential biomarkers for obesity, but their roles in obesity may extend beyond previous findings, potentially involving interactions with host genetics.

Food-induced obesity could decrease the abundance of *g_Turicibacter*, which is known to play a role in the 5-hydroxytryptamine signaling. *g_Turicibacter* may exert potential protective effects against colorectal tumorigenesis by enhancing the Wnt signaling pathway and inducing ROS-mediated cell apoptosis [105]. In this study, a significant decrease in the abundance of *g_Turicibacter* was observed in obese children, suggesting a potential reduction in its beneficial effects on the human body. Moreover, *g_Turicibacter* occupies a significant portion of the interactome network and is significantly associated with DEMs such as Tetradecanedioic acid, Lithocholic acid, and Acamprosate. Investigating the potential causal relationship between *g_Turicibacter* and these metabolites may help elucidate the crosstalk between the gut microbiota and host metabolism in obesity.

Previous studies have suggested that *p_Firmicutes* can serve as a biomarker for obesity. In this study, the AUC of *p_Firmicutes* alone was only 66.5%, while the combined AUC area of *p_Firmicutes*, *c_Negativicutes*, *o_Selenomonadales*, and *f_Veillonellaceae* reached 81.7%. Therefore, we believe that combining these four microbiotas may provide better predictive value for the diagnosis of obesity. A prospective study found that levels of *f_Veillonellaceae* in the gut significantly increased in Class II and Class III obese patients after weight-loss surgery, often observed after substantial weight loss, and associated with improvements in metabolic characteristics. These changes could be explained by factors other than dietary changes, such as gastrointestinal rearrangement, determinants of bile acid production, and alterations in luminal pH. *o_Selenomonadales* ferment carbohydrates into acetate and lactate, which are associated with the formation of short-chain fatty acids (SCFAs) [106], and have been implicated in Alzheimer’s disease [107], idiopathic nephrotic syndrome [108], and identified as a risk factor for insomnia. *c_Negativicutes*, a gram-negative bacterium, serves as a biomarker for Class III obesity [109], enriched in late-stage NAFLD or cirrhosis patients [5, 110, 111], and exhibits a causal relationship with insomnia [112]. Additionally, functional enrichment analysis of gut microbiota in obesity revealed significant upregulation in amino acid transport and metabolism, while downregulation in energy production and conversion, lipid transport and metabolism. Microbial enrichment identified between the two groups was significantly enriched in the Metabolism of cofactors and vitamins, Metabolism of terpenoids and polyketides, as suggested by LEfSe, highlighting significant enrichment in the Phosphotransferase system, Porphyrin and chlorophyll metabolism, Glycerolipid metabolism. Although there have been numerous studies on gut microbiota-host metabolism interactions in recent years, they have mainly focused on the impact of gut microbiota on fat and sugar metabolism, neglecting cofactors, vitamins, terpenoids and polyketides, and porphyrin and chlorophyll metabolism. Further research in these areas may provide novel insights and approaches for the diagnosis and treatment of obesity.

In the process of integrating multi-omics correlation analysis, we identified a set of biomarkers with well-interconnected networks implicated in obesity, such as BPIFA1, BPI, SAA1, PDE1C, Deoxycholic acid, Phthalic anhydride, DL-Alanine, *p_Firmicutes*, *g_Intestinimonas*, and *g_Turicibacter*. In the future, our team will further investigate these potential biomarkers and their interactions and/or causal relationships, particularly focusing on validating the following relationship: PDE1C = -0.004* *p_Firmicutes* + 3.059DL-Glutamine − 571.6Epiandrosterone + 206.5*3-Hydroxypicolinic-acid. We aim to provide novel perspectives and strategies for understanding the pathogenesis, prevention, and treatment of obesity.

## Conclusions

This study provides novel, multi-layered insights into the pathogenesis of childhood simple obesity. Identified differential immune genes, metabolites, and gut microbiota hold promise as biomarkers for childhood simple obesity. Revealing their interrelationships contributes to a better understanding of the intricate regulatory networks of biological systems in childhood simple obesity.

## Electronic supplementary material

Below is the link to the electronic supplementary material.

Supplementary Material 1

Supplementary Material 2

Supplementary Material 3

Supplementary Material 4

Supplementary Material 5

Supplementary Material 6

Supplementary Material 7

Supplementary Material 8

Supplementary Material 9

## Data Availability

The data obtained in the analysis of this article are included in this paper, and the raw data reported here are available upon request to the corresponding authors.

## Abbreviations

AUC: Area under the ROC curve
BAT: Brown adipose tissue
BMI: Body mass index
COG: Cluster of Ortholog Genes
DAMPs: Disease-associated molecular patterns
DEGs: Differentially expressed genes
DEMs: Differentially expressed metabolites
DGMs: Differential gut microbiotas
FC: Fold-change
FPKM: Fragments Per Kilobase of exon model per Million mapped reads
GO: Gene Ontology
HFD: High-fat diet
IFN: Interferon
ILA: Indole-3-lactic acid
iWAT: White adipose tissue
KEGG: Kyoto Encyclopedia of Genes and Genomes
LA: Lauric acid
LCN2: Lipocalin-2
LDA: Linear discriminant analysis
LEfSe: LDA effect size
NAFLD: Nonalcoholic fatty liver disease
OA: Oleic acid
OEA: Oleoylethanolamide
OPLS: DA-Orthogonal PLS-DA
OTUs: Operational Taxonomic Units
PAR3: Protease-activated receptor 3
PCoA: Principal coordinate analysis
PLS: DA-Partial Least Squares Discriminant analysis
PPI: Protein-Protein Interaction
QC: Quality control
ROC: Receiver-operating characteristic
SCFAs: Short-chain fatty acid
T2DM: Type 2 diabetes mellitus
TG: Triglycerides
TGR5: Transmembrane G protein-coupled bile acid receptor 5 (TGR5)
TMM: Trimmed Mean of M
UA: Uric acid
Vit D: Vitamin D
WAT: White adipose tissue
