# Supplementary material for: Multi-omics landscape of childhood simple obesity: novel insights into pathogenesis and biomarkers discovery
Source: Cell Biosci. 2024 Nov 28;14:145. doi: 10.1186/s13578-024-01322-5 (PMC11606102; doi:10.1186/s13578-024-01322-5)
Supplement: Supplementary file 1 — Supplementary Material 1 [file 13578_2024_1322_MOESM1_ESM.docx]

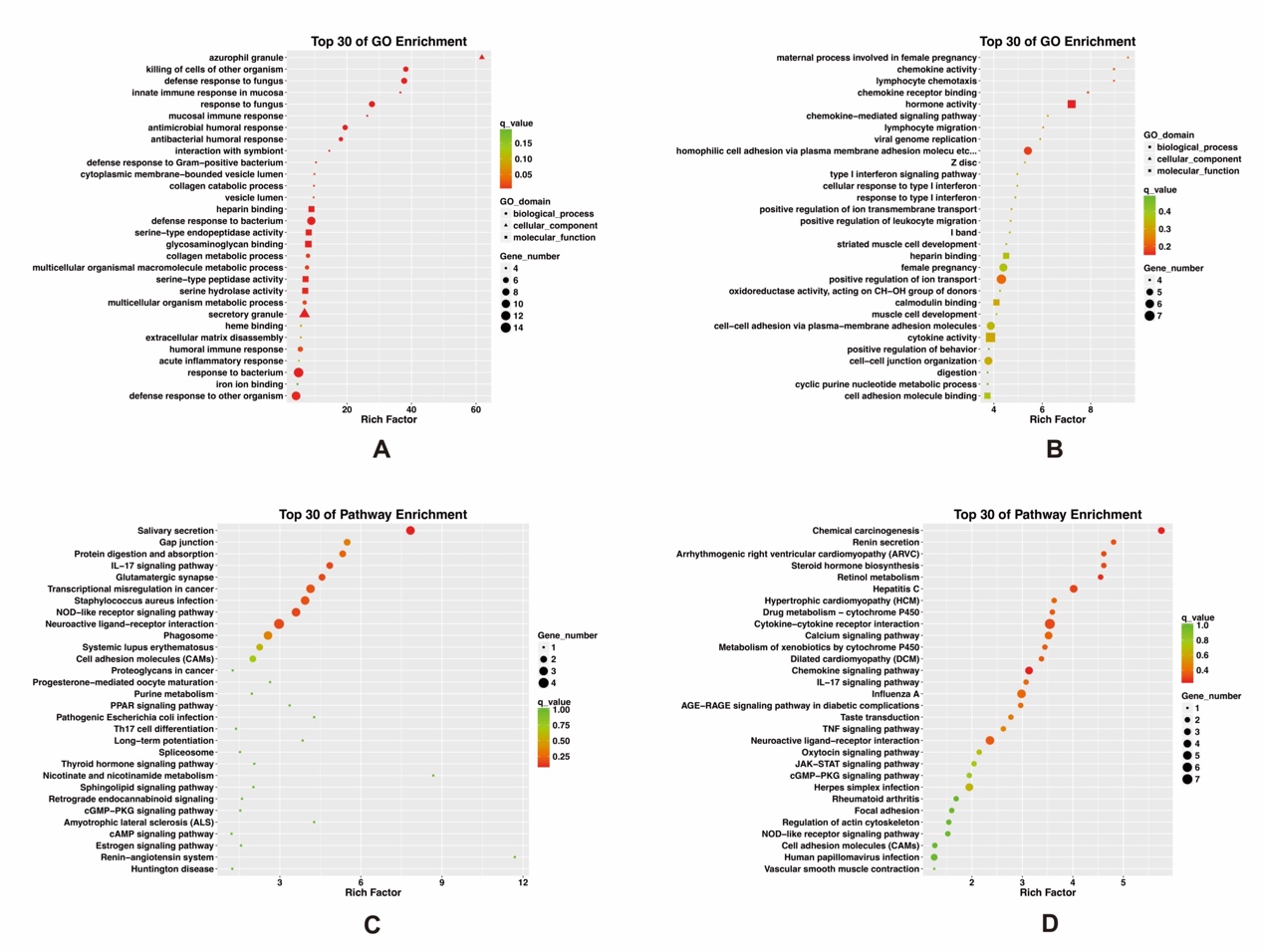


**Fig. S1.** Gene enrichment analysis of upregulated and downregulated DEGs. (A) and (B) are bubble plots of GO enrichment analysis for upregulated and downregulated DEGs, respectively, showing the top 30 enriched GO terms. (C) and (D) are bubble plots of KEGG enrichment for upregulated and downregulated DEGs, respectively, showing the top 30 enriched KEGG pathways. A larger Rich Factor and smaller *q*-value indicate a higher enrichment level. Larger bubbles indicate more enriched genes.


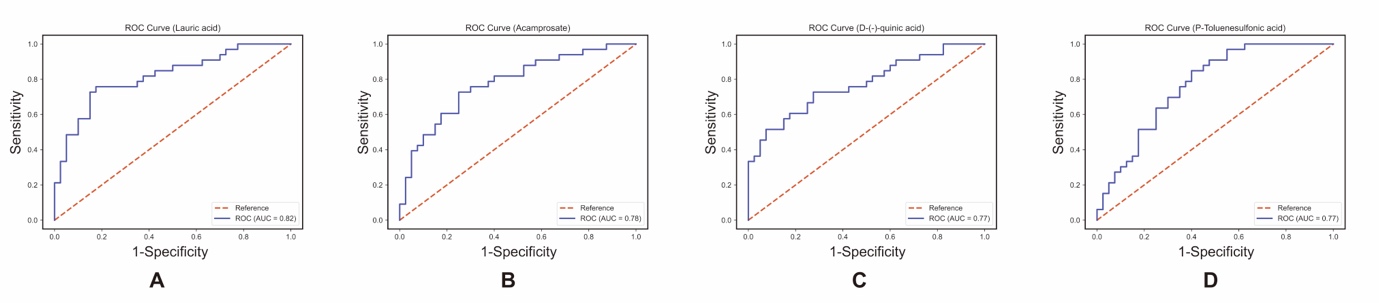


**Fig. S2.** AUC for differential metabolites Lauric acid, Acamprosate, P-Toluensulfonic, and D-(-)-quinic acid, respectively.


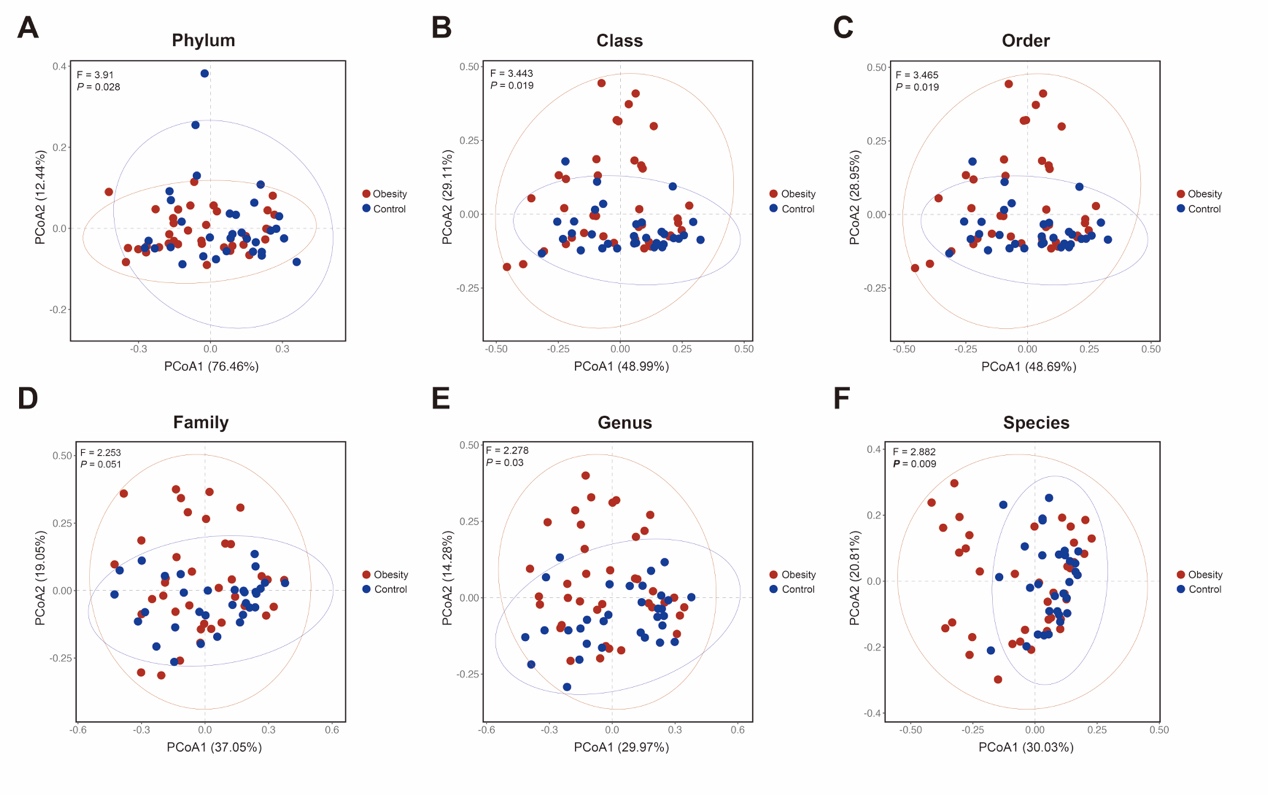


**Fig. S3.** Beta diversity comparison of gut microbiota between two groups at different taxonomic levels (Phylum, Class, Order, Family, Genus, and Species) based on PERMANOVA analysis.
